# Exosomal Long Noncoding RNA H19 as a Biomarker and Therapeutic Target in Atrial Fibrillation

**DOI:** 10.7150/ijbs.123108

**Published:** 2026-03-17

**Authors:** Ji-Young Kang, Dasom Mun, Malgeum Park, Gyeongseo Yoo, Nuri Yun, Boyoung Joung

**Affiliations:** 1 Division of Cardiology, Yonsei University College of Medicine, 50-1 Yonsei-ro, Seodaemun-gu, Seoul 03722, Republic of Korea.; 2 Graduate School of Medical Science, Brain Korea 21 Project, Yonsei University College of Medicine, 50-1 Yonsei-ro, Seodaemun-gu, Seoul 03722, Republic of Korea.; 3 GNTPharma Science and Technology Center for Health, 85 Songdogwahak-ro, Yeonsu-gu, Incheon 21983, Republic of Korea.

**Keywords:** atrial fibrillation, exosomes, long noncoding RNAs, biomarker, therapeutic target

## Abstract

Although exosomal long noncoding RNAs (lncRNAs) have emerged as promising theragnostic targets in various diseases, their role in atrial fibrillation (AF) remains largely unexplored. Herein, we aimed to identify AF-specific serum exosomal lncRNAs and to evaluate their potential as theragnostic targets. RNA sequencing and qRT-PCR analyses consistently demonstrated significant downregulation of lncRNA *H19* in serum exosomes of patients with AF compared with those without AF. Notably, serum exosomal lncRNA *H19* levels showed significant diagnostic validity for AF and were closely associated with AF pathophysiology. In angiotensin II (Ang II)-treated iPSC-derived atrial cardiomyocytes, both loss- and gain-of-function experiments revealed that lncRNA *H19* markedly modulated Ang II-induced hypertrophic responses, including increased expression of *ANP*, *BNP*, and *β-MHC*, as well as enlargement of cell surface area. Moreover, *in vivo* experiments showed that cardiac-specific overexpression of lncRNA *H19* significantly attenuated Ang II-induced cardiac dysfunction and hypertrophy (*P* < 0.05). Mechanistically, lncRNA *H19* sponges *miR-141-3p* and *miR-200a-3p*, thereby regulating the PTEN pathway and contributing to cardiac hypertrophic remodeling and subsequent AF. Collectively, these findings identify a novel association between circulating exosomal lncRNA *H19* and AF and further elucidate its mechanistic role in cardiac hypertrophy, highlighting its potential as a diagnostic biomarker and therapeutic target for AF.

## Introduction

Atrial fibrillation (AF) is the most common arrhythmia and is increasingly recognized as a 21st-century cardiovascular epidemic due to its rising global incidence 1-3. AF increases mortality and morbidity from stroke, dementia, and heart failure, imposing a high healthcare burden 4-6. Despite the latest advancements, effective medications or procedures that conclusively reduce AF or prevent its pathogenesis remain lacking 1, 4. Therefore, the development of novel therapeutic approaches that could facilitate timely detection and treatment of AF is crucial.

Long noncoding RNAs (lncRNAs)—noncoding transcripts longer than 200 nucleotides—represent a new layer of gene regulatory networks 7, 8. LncRNAs are widely expressed and play important roles in biological functions and pathological processes of various diseases, including cardiovascular diseases 9, 10. Notably, lncRNAs affect critical pathways and core proteins related to AF pathophysiology 11, 12. For instance, it has been demonstrated that lncRNA *NEAT1* regulates atrial fibrosis through the miRNA-320/NPAS2 axis 13. In addition, lncRNA *TCONS-00106987* has been reported to promote atrial electrical remodeling during AF through the miRNA-26/KCNJ2 axis 14. Consequently, lncRNAs have garnered increasing attention as key regulators in AF pathogenesis 15, 16. Nevertheless, most studies have focused on intracellular lncRNAs, and the roles of circulating or exosome-associated lncRNAs in AF remain poorly understood.

Exosomes are small membrane-bound organelles secreted by all cell types into the extracellular fluids, including peripheral blood (serum/plasma), urine, and saliva 17. Exosomes play important roles in mediating cell-cell communication by transferring a variety of molecules such as proteins, lipids, and nucleic acids 18, 19. Exosomes are surrounded by a lipid bilayer membrane that protects their molecular content from degradation in circulation 20, 21. Interestingly, circulating lncRNAs are usually encapsulated within exosomes and exhibit different expression profiles in various diseases 22, 23. Therefore, exosomal lncRNAs have attracted considerable attention as potential noninvasive diagnostic biomarkers.

Exosomal lncRNAs are also regarded as promising therapeutic targets for various diseases 20, 23. Notably, the molecular content of exosomes is largely influenced by a particular medical condition, reflecting the different pathophysiological states of parental cells 20, 24. For instance, it has been demonstrated that dysregulation of lncRNA *THEMIS2-211* in both tissues and exosomes promotes tumor growth and metastasis in hepatocellular carcinoma, emphasizing disease-dependent alterations of exosomal lncRNAs as promising theragnostic targets 25. However, only a few studies have investigated specific changes in exosomal lncRNAs and their clinical relevance in AF.

In the present study, we identified specific serum exosomal lncRNAs in patients with AF and investigated their functional roles in AF pathogenesis. Circulating exosomes from patients with AF contained lower lncRNA *H19* levels than those from patients without AF, indicating its potential as a molecular signature of AF. Moreover, we elucidated the potential molecular mechanism by which lncRNA *H19* deficiency promotes cardiac hypertrophy through the lncRNA-miRNA-mRNA regulatory network. Therefore, our study highlights new potential biomarkers and therapeutic targets for the prevention and treatment of AF and its associated complications.

## Materials and Methods

### Clinical samples

Human blood samples were obtained from controls (n=68) and patients diagnosed with persistent AF (n=68), all of whom underwent radiofrequency (RF) catheter ablation at the Yonsei University Health System (Seoul, South Korea). Patients with paroxysmal supraventricular tachycardia were classified as controls; none had tachycardia for at least one week prior to blood sampling to minimize tachycardia-induced changes in lncRNA expression. All blood samples were collected with written informed consent before RF ablation using BD Vacutainer® Serum Tubes (367815; Becton Dickinson, Franklin Lakes, NJ, USA). Serum was separated by centrifugation at 1,500 × *g* for 10 min and stored at -80 °C for subsequent experiments. Atrial appendage tissues were obtained from patients with sinus rhythm (n=8) and persistent AF (n=8) during open-heart surgery at the Yonsei University Health System with written informed consent. The clinical characteristics of all participants are summarized in Table S1 and Table S2. This study was approved by the Institutional Review Board of Severance Hospital, Yonsei University Health System (Seoul, South Korea; approval numbers 4-2011-0872 and 4-2019-0620) and was conducted in accordance with the Declaration of Helsinki.

### Cell culture and angiotensin II (Ang II) treatment

HEK293 cells were obtained from the Korean Cell Line Bank (Seoul, South Korea) and cultured in Dulbecco's modified Eagle medium (LM001-05; Welgene, Gyeongsan, South Korea) supplemented with 10% fetal bovine serum (US-FBS-500; Young In Frontier, Seoul, South Korea) and 1% penicillin-streptomycin (10378016; Gibco, Grand Island, NY, USA).

The human induced pluripotent stem cell (iPSC) line CMC-hiPSC-011 was obtained from the Korea National Stem Cell Bank, originally provided by Catholic University. iPSCs were differentiated into ventricular cardiomyocytes (iPSC-vCMs) and atrial cardiomyocytes (iPSC-aCMs), as previously described 26, 27. When iPSCs reached 80%‒90% confluence, differentiation was initiated on Matrigel (354277; Corning, Glendale, AZ, USA)-coated plates using RPMI 1640 (11875119; Thermo Fisher Scientific, Waltham, MA, USA) supplemented with B-27 minus insulin (A1895601; Thermo Fisher Scientific), followed by sequential treatment with 2 µM CHIR99021 (4423; Tocris Bioscience, Bristol, UK) for 72 h and 2 µM Wnt-C59 (5148; Tocris Bioscience) for 48 h. On day 9, the medium was replaced with RPMI 1640 supplemented with B-27 (17504001; Thermo Fisher Scientific). For atrial subtype differentiation, retinoic acid (1 μM) (R2625; Sigma-Aldrich, Merck KGaA, Darmstadt, Germany) was supplemented from days 4 to 7.

To induce cardiac hypertrophy *in vitro*, iPSC-aCMs were treated with 2.5 μM Ang II (A9525; Sigma-Aldrich, Merck KGaA) for 24 h, which was applied in all subsequent functional experiments. Cells were maintained at 37 °C in a humidified incubator (Thermo Fisher Scientific) with 5% CO_2_. All cell lines were routinely tested for mycoplasma contamination and confirmed to be mycoplasma-free.

### Isolation and characterization of exosomes

Human serum-derived exosomes were isolated using Total Exosome Isolation (from serum) Reagent (4478360; Thermo Fisher Scientific) according to the manufacturer's protocol. Briefly, human serum samples were centrifuged at 2,000 × *g* for 30 min to remove cells and debris. The supernatant (500 μL) was then mixed with Total Exosome Isolation (from serum) Reagent and incubated for 30 min at 4 °C, followed by centrifugation at 10,000 × *g* for 10 min at room temperature. In addition, exosomes derived from iPSC-aCMs-conditioned culture medium were isolated using Total Exosome Isolation (from cell culture media) Reagent (4478359; Thermo Fisher Scientific) according to the manufacturer's protocol. Exosome pellets were resuspended in 200 μL of Dulbecco's phosphate-buffered saline (DPBS), aliquoted, and stored at -80 °C for subsequent experiments.

For ultrastructural analysis, exosome suspensions were deposited onto Formvar carbon-coated copper grids (Leica Microsystems, Inc., Buffalo Grove, IL, USA) and negatively stained with 2% uranyl acetate. Exosome morphology was subsequently examined using transmission electron microscopy (JEM-1011; JEOL Ltd., Tokyo, Japan).

Particle size distribution and concentration were determined using nanoparticle tracking analysis (NTA) on a NanoSight LM10 instrument (Malvern Instruments Ltd, Malvern, UK). Exosome Brownian motion was recorded for 60 s at room temperature and analyzed using NTA software (version 2.3; Malvern Panalytical, Ltd.) based on the Stokes‒Einstein equation.

### Cell transfection

Synthetic miRNAs and siRNAs, including negative control (NC) miRNA, *miR-141-3p* mimic, *miR-141-3p* inhibitor, *miR-200a-3p* mimic, *miR-200a-3p* inhibitor, NC siRNA (siNC), lncRNA *H19* siRNA (siH19), and *PTEN* siRNA (siPTEN), were obtained from Bioneer (Daejeon, South Korea). The NC and lncRNA *H19* overexpression vectors were constructed by VectorBuilder Inc. (Chicago, IL, USA). When iPSC-aCMs reached 60%‒80% confluence, cells were transfected using Lipofectamine™ RNAiMAX (13778150; Thermo Fisher Scientific) or Lipofectamine™ 3000 (L3000001; Thermo Fisher Scientific) according to the manufacturers' protocols.

### Luciferase reporter assay

The 3′-untranslated region (UTR) fragments of lncRNA *H19* or *PTEN* containing the predicted wild-type (WT) or mutated (Mut) binding sites for *miR-141-3p* and *miR-200a-3p* were amplified and inserted into pmirGLO Vector (E1330; Promega, Madison, WA, USA). HEK293 cells were selected because of their high transfection efficiency and reliable performance in reporter-based analyses. Cells were cultured in 24-well plates and transfected with the corresponding plasmids, *miR-141-3p* mimic, *miR-200a-3p* mimic, or NC miRNA using Lipofectamine™ 3000 (L3000001; Thermo Fisher Scientific). Forty-eight hours after transfection, luciferase activity was measured using Dual-Glo Luciferase Assay System (E2920; Promega) according to the manufacturer's protocol.

### Pull-down assay with biotinylated miRNAs

HEK293 cells were seeded in 24-well plates for 24 h and transfected with biotin-labeled NC (Bio-NC), biotin-labeled WT-*miR-141-3p* (Bio-WT-141), biotin-labeled Mut-*miR-141-3p* (Bio-Mut-141), biotin-labeled WT-*miR-200a-3p* (Bio-WT-200a), or biotin-labeled Mut-*miR-200a-3p* (Bio-Mut-200a). All biotin-labeled miRNAs were synthesized by Bioneer (Daejeon, South Korea), and the sequences are listed in Table S3. At 48 h post-transfection, cells were harvested and lysed on ice for 10 min in lysis buffer containing 20 mM Tris-HCl (pH 7.5, 15567027; Thermo Fisher Scientific), 200 mM NaCl (S9888; Sigma-Aldrich, Merck KGaA), 2.5 mM MgCl_2_ (M8266; Sigma-Aldrich, Merck KGaA), 0.05% IGEPAL CA-630 (I8896; Sigma-Aldrich, Merck KGaA), 60 U ml^-1^ SUPERase-In RNase inhibitor (AM2694; Thermo Fisher Scientific), 1 mM DTT (707265ML; Thermo Fisher Scientific), and protease inhibitor cocktail (11836170001; Sigma-Aldrich, Merck KGaA). The lysates were incubated with streptavidin magnetic beads (Dynabeads^TM^ M-280 Streptavidin, 11205D; Thermo Fisher Scientific) pre-blocked with bovine serum albumin (BSA) (AM2616; Thermo Fisher Scientific) and yeast tRNA (AM7119; Thermo Fisher Scientific) to minimize non-specific binding. After incubation at 4 °C for 3 h, the beads were washed twice with ice-cold lysis buffer, three times with low salt wash buffer (BL012; Biosolution, Gyeonggi-do, South Korea), and once with high salt wash buffer (BH032; Biosolution). The enriched lncRNA *H19* levels in the pulldown samples were analyzed by quantitative reverse transcription polymerase chain reaction (qRT-PCR).

### Evaluation of cell size

iPSC-aCMs were fixed with 4% paraformaldehyde and permeabilized using 0.3% Triton X-100/PBS. After blocking with 1% BSA/PBS, cells were incubated overnight at 4 °C with a primary antibody against α-actinin (A7811, 1:200; Sigma-Aldrich, Merck KGaA). Following three washes with PBS, cells were exposed to a fluorophore-conjugated secondary antibody (4408S, 1:1000; Cell Signaling Technology, Danvers, MA, USA) for 1 h at room temperature. Cell nuclei were counterstained with Hoechst 33342 (H3570; Thermo Fisher Scientific). Immunofluorescence images were obtained using confocal microscopy (Zeiss LSM 710; Carl Zeiss, Oberkochen, Germany), and cell surface area was quantified using ImageJ software (version 1.50i; National Institutes of Health, Bethesda, MD, USA).

### qRT-PCR

Cellular or exosomal RNA was extracted using miRNeasy^®^ Mini Kit (217004; Qiagen, Hilden, Germany). Each RNA sample was reverse-transcribed into complementary DNA (cDNA) using High-Capacity cDNA Reverse Transcription Kit (4368814; Applied Biosystems, Darmstadt, Germany) and miRNA 1st-Strand cDNA Synthesis Kit (600036; Agilent Technologies, Santa Clara, CA, USA) according to the manufacturers' protocols. qRT-PCR was performed using PowerUp™ SYBR™ Green Master Mix (A25742; Applied Biosystems) on an AriaMx Real-time PCR System (Agilent Technologies). All primers were synthesized by Cosmo Genetech (Daejeon, South Korea), and the sequences are listed in Table S4. Relative expression levels of mRNAs and miRNAs were calculated using the 2^-ΔΔCq^ method and normalized to *GAPDH* and *U6*, respectively 28.

### Western blotting

Protein lysates were prepared in radioimmunoprecipitation assay buffer containing protease and phosphatase inhibitor cocktails (WSE-7420; ATTO, Tokyo, Japan), followed by protein quantification using the Pierce™ 660 nm Protein Assay Reagent (22660; Thermo Fisher Scientific). Quantified protein samples were separated by 10% SDS-polyacrylamide gel electrophoresis and transferred to polyvinylidene fluoride membranes (IPVH00010; EMD Millipore, Bedford, MA, USA). To block nonspecific binding, membranes were incubated for 1 h at room temperature in 5% BSA/Tris-buffered saline containing 0.1% Tween 20. The membranes were then incubated overnight at 4 °C with primary antibodies against TSG101 (sc-7964, 1:1,000; Santa Cruz Biotechnology Inc., Dallas, TX, USA), CD81 (sc-166029, 1:1,000; Santa Cruz Biotechnology Inc.), GRP94 (sc-393402, 1:1,000; Santa Cruz Biotechnology Inc.), cTnI (ab47003, 1:1,000; Abcam, Cambridge, UK), MYL2 (sc-517244, 1:1,000; Santa Cruz Biotechnology Inc.), PTEN (9559S, 1:1,000; Cell Signaling Technology), AKT (9272S, 1:1,000; Cell Signaling Technology), p-AKT (9271S, 1:1,000; Cell Signaling Technology), and β-actin (sc-47778, 1:1,000; Santa Cruz Biotechnology Inc.). After exposure to horseradish peroxidase-conjugated secondary antibodies (sc-516102 or sc-2357, 1:5,000; Santa Cruz Biotechnology Inc.) for 1 h at room temperature, immunoreactive signals were detected using an enhanced chemiluminescence kit (1705061; Bio-Rad Laboratories Inc., Hercules, CA, USA). All Western blot analyses were performed using at least three independent biological replicates. Band intensities were quantified using ImageJ software (version 1.50i; National Institutes of Health), normalized to β-actin, and are presented as relative densitometric values.

### RNA sequencing

The integrity of exosomal RNA from six serum samples (three controls and three patients with AF) was assessed using an Agilent Technologies 2100 Bioanalyzer. Libraries were constructed using the SMARTer smRNA-Seq Kit (Illumina, San Diego, CA, USA) and single-end RNA-sequenced on an Illumina HiSeq 2500 platform. The quality of the raw FASTQ files was evaluated using FastQC (version 0.11.7; https://sourceforge.net/projects/fastqc.mirror/files/v0.11.7/), and reads were trimmed using Cutadapt (version 2.8; https://cutadapt.readthedocs.io/en/v2.8/). To classify known miRNAs and other RNA species, the processed reads from each sample were sequentially aligned to the human reference genome (GRCh38), miRBase release 22.1, and a noncoding RNA database (RNAcentral version 14.0). After mapping and counting, differential expression analysis was conducted using the DESeq R package (version 3.5.3; Lucent Technologies, New Providence, NJ, USA). All RNA sequencing-related procedures were performed by Macrogen (Seoul, South Korea).

### Identification and functional analysis of target genes

The DAVID database (https://david.ncifcrf.gov/) was used to perform Gene Ontology (GO) enrichment analysis. GO biological process, cellular component, and molecular function terms with an adjusted *P* < 0.05 were considered significantly enriched. Core pathway analysis was conducted using Ingenuity Pathway Analysis software (Qiagen). The CytoHubba plug-in of Cytoscape (version 3.10.2; https://manual.cytoscape.org/en/3.10.2/) was used to identify hub genes within the protein-protein interaction (PPI) network.

### Animal experiments

All animal procedures were approved by the Institutional Animal Care and Use Committee of Yonsei University College of Medicine (approval number 2023-0252) and were conducted in compliance with the Guide for the Care and Use of Laboratory Animals published by the US National Institutes of Health (Publication number 85-23, revised 1996). Male C57BL/6 mice (7 weeks old) were purchased from Orient Bio Inc. (Seongnam, South Korea) and housed under controlled environmental conditions (temperature, 20 ± 0.5 °C; humidity, 60% ± 5%; 12 h light/dark cycle) with *ad libitum* access to food and water. To establish an *in vivo* AF model, mice were anesthetized by intraperitoneal injection of tiletamine-zolazepam (Zoletil 50, 30 mg/kg; Virbac, Carros, France) and xylazine (Rompun^®^, 10 mg/kg; Bayer, Seoul, South Korea), and Ang II was continuously infused via subcutaneous implantation of Ang II-containing Alzet^®^ 1002 micro-osmotic pumps (1.5 mg/kg/day; Durect Corp., Cupertino, CA, USA). Control mice were implanted with pumps containing PBS. For cardiac-specific overexpression of lncRNA *H19*, an adeno-associated virus serotype 9 (AAV9) harboring either the lncRNA *H19* sequence under the control of chicken cardiac troponin T (cTnT) promoter (AAV9-H19) or an empty control vector (AAV9-Con) was constructed by VectorBuilder Inc. and administered via intravenous injection 7 days prior to pump implantation. Mice were randomly assigned to experimental groups (n=4 per group). Two weeks after implantation, mice were euthanized and cardiac tissues were quickly harvested for subsequent experiments.

Cardiac function was assessed using echocardiography on a Vevo 2100 system (VisualSonics, Toronto, Ontario, Canada). Left ventricular ejection fraction (LVEF) and left ventricular fractional shortening (LVFS) were calculated as follows:

LVEF (%) = [(LV Vol;d - LV Vol;s) / LV Vol;d] × 100; LVFS (%) = [(LVID;d - LVID;s) / LVID;d] × 100

where LV Vol;d is the left ventricle volume diastole, LV Vol;s is the left ventricle volume systole, LVID;d is the left ventricular internal diameter (diastole), and LVID;s is the left ventricular internal diameter (systole).

For histological analysis, cardiac tissues were fixed in 4% paraformaldehyde, embedded in paraffin, and sectioned at a thickness of 4 μm. The sections were stained with hematoxylin and eosin (H&E) and Alexa Fluor 488-conjugated wheat germ agglutinin (W11261; Thermo Fisher Scientific). In addition, diaminobenzidine (DAB) staining was performed using an antibody against PTEN (9559S; Cell Signaling Technology). Stained sections were examined using an inverted microscope (Olympus, Tokyo, Japan) or a confocal microscope (Zeiss LSM 710; Carl Zeiss). Four hearts were analyzed per experimental group, and three individual fields were evaluated from each section, resulting in a total of 12 fields per group. Quantitative image analysis was performed using ImageJ software (version 1.50i; National Institutes of Health).

To assess *in vivo* systemic toxicity, serum levels of biochemical parameters, including alanine aminotransferase (ALT), aspartate aminotransferase (AST), blood urea nitrogen (BUN), creatinine (CRE), and lactate dehydrogenase (LDH), were quantified using Fuji Dri-Chem 4000i (Fujifilm, Tokyo, Japan) according to the manufacturer's protocol.

### Statistical analyses

Data are presented as the mean ± standard deviation. Comparisons between two groups and multiple groups were performed using a two-tailed Student's *t*-test and one-way analysis of variance (ANOVA) with Tukey's post-hoc test, respectively. Logistic regression analysis was performed to calculate adjusted odds ratio (OR) for exosomal lncRNA *H19* after adjustment for clinical variables, including age, sex, body mass index (BMI), coronary artery disease (CAD), diastolic blood pressure (BP), left atrial diameter (LAD), and LVEF. Receiver operating characteristic (ROC) curve analysis was conducted to determine the area under the curve (AUC), sensitivity, and specificity of exosomal lncRNA *H19*. Sample size estimation was performed using G*Power software (version 3.1.9.7; Heinrich-Heine-Universität Düsseldorf, Germany) 29. All data were analyzed using SPSS software (version 26; IBM SPSS Inc., Chicago, IL, USA) and GraphPad Prism (version 10.5.0; GraphPad, San Diego, CA, USA). A *P* < 0.05 was considered statistically significant.

## Results

### Serum exosomal lncRNA *H19* acts as a new target for AF diagnosis

**Figure 1:**
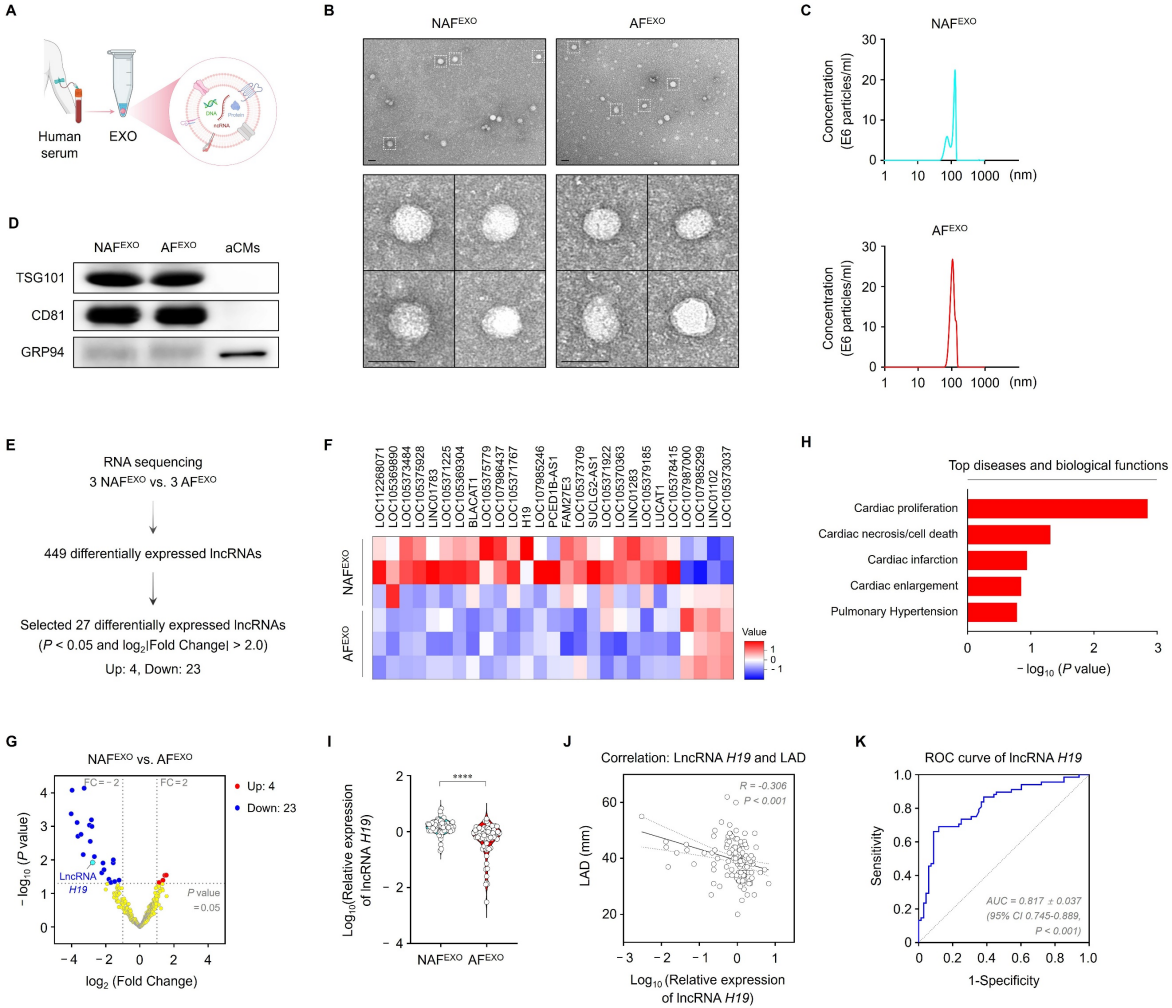
Identification of serum exosomal lncRNA *H19* as a biomarker for AF. (A) Schematic illustration of exosome isolation from human serum. (B) Transmission electron micrographs of NAF^EXO^ and AF^EXO^. Scale bar = 100 nm. (C) Size distribution of NAF^EXO^ and AF^EXO^. (D) Representative blots of TSG101, CD81, and GRP94 in iPSC-aCMs lysates and both types of exosomes. Experiments were performed using at least three independent biological replicates. Uncropped blots are presented in Figure S16. (E) Schematic illustration of RNA sequencing profiles. (F) Heat map of 27 differentially expressed serum exosomal lncRNAs between AF^EXO^ (n = 3) and NAF^EXO^ (n = 3). Red indicates relatively higher expression, whereas blue indicates relatively lower expression. (G) Volcano plot for the comparison of lncRNA expression profiles between NAF^EXO^ and AF^EXO^. X-axis, differential expression profiles, plotting the log_2_ (fold change); Y-axis, statistical significance of differences. (H) Top diseases and biological functions of lncRNA *H19* identified using Ingenuity Pathway Analysis. (I) qRT-PCR analysis of lncRNA *H19* levels in the indicated groups (n = 68 per group). Data are normalized to *GAPDH* levels. (J) Correlation analysis of serum exosomal lncRNA *H19* levels with LAD. (K) ROC curve analysis for prediction of AF by serum exosomal lncRNA *H19*. ****P < 0.0001. NAF^EXO^, serum-derived exosomes from patients without AF; AF^EXO^, serum-derived exosomes from patients with AF; LAD, left atrial diameter; ROC, receiver operating characteristic; AUC, area under the curve; CI, confidence interval.

We first isolated serum-derived exosomes from patients with AF (AF^EXO^) and those without AF (NAF^EXO^) using a polymer-based precipitation method (**Figure 1A**). Transmission electron micrographs revealed that the isolated exosomes exhibited a typical round, cup-shaped morphology with a mean diameter of ~150 nm (**Figure 1B**). NTA demonstrated no significant differences in size distribution or concentration between NAF^EXO^ and AF^EXO^ (**Figure 1C**). Western blot analysis confirmed the enrichment of exosome marker proteins TSG101 and CD81 in both exosome types, whereas GRP94, which is expressed in the endoplasmic reticulum, was not detected in the exosomes (**Figure 1D**), indicating successful isolation of exosomes from human serum.

To identify serum exosomal lncRNAs with diagnostic potential for AF, lncRNA expression profiles of NAF^EXO^ and AF^EXO^ were analyzed using RNA sequencing. A total of 27 differentially expressed lncRNAs (i.e., 4 upregulated and 23 downregulated) were detected in AF^EXO^ compared with NAF^EXO^ (filtering criteria, |fold change| ≥ 2; *P* < 0.05) (**Figure 1E-G**) and are listed in **Table S5**. Among the 27 lncRNAs, lncRNA *H19* stood out as a potential candidate because it is involved in multiple AF pathophysiological processes, including cardiac proliferation, necrosis/cell death, and enlargement (**Figure 1H**). The remaining lncRNAs have not been functionally characterized or showed limited associations with AF pathophysiology. We then verified the differential expression of lncRNA *H19* between NAF^EXO^ and AF^EXO^. The levels of serum exosomal lncRNA *H19* decreased by approximately 2.9-fold in patients with AF compared with those without AF (*P* < 0.05) (**Figure 1I**). As shown in **Figure 1J**, serum exosomal lncRNA *H19* levels were negatively correlated with LAD (*r* = -0.306; *P* < 0.001). Moreover, ROC curve analysis demonstrated an AUC of 0.817 ± 0.037 (95% confidence interval [CI], 0.745-0.889; *P* < 0.001) (**Figure 1K**). Furthermore, multivariate logistic regression analysis showed that, after adjustment for age, sex, and baseline clinical variables, reduced serum exosomal lncRNA *H19* levels were independently associated with AF (OR=0.04, 95% CI < 0.01-0.35, *P* < 0.001) (**Table S6**). Collectively, these findings demonstrate specific changes in serum exosomal lncRNA *H19* in patients with AF, indicating its potential as a diagnostic biomarker for AF.

### LncRNA *H19* plays a role in cardiac hypertrophy

Cardiac hypertrophy is an important risk factor for AF development 30. Although cardiac hypertrophy initially represents a compensatory response to maintain cardiac function, prolonged hypertrophy leads to progressive structural and electrical remodeling and impaired cardiac function, eventually promoting AF 31, 32. Therefore, prevention of cardiac hypertrophy is considered a potential upstream therapeutic strategy for AF, emphasizing the need to investigate the role of lncRNA *H19* in cardiac hypertrophy.

**Figure 2:**
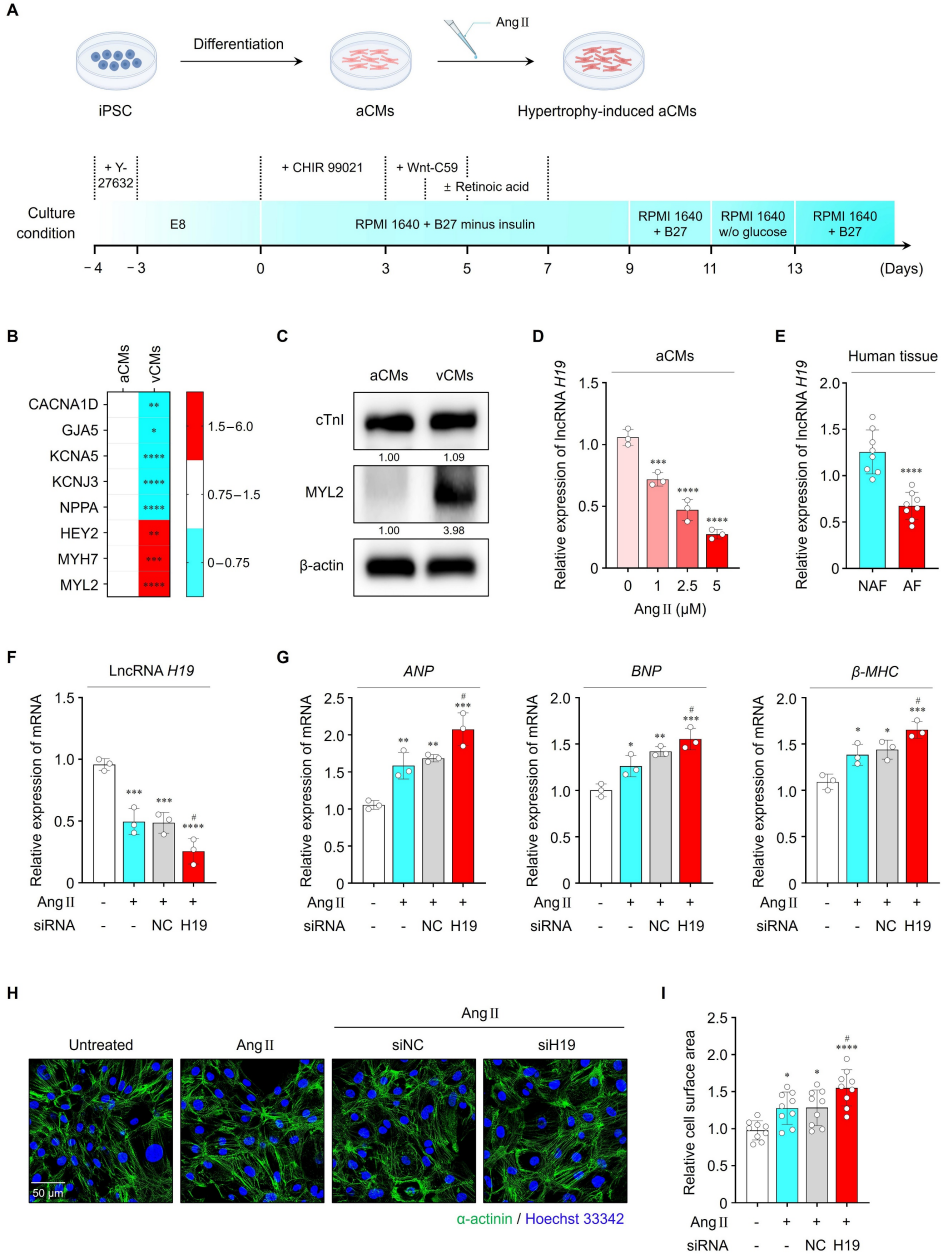
Effects of lncRNA *H19* on cardiac hypertrophy. (A) Schematic illustration of iPSC-aCMs differentiation protocol. (B) qRT-PCR analysis of atrial- or ventricular-specific gene levels in iPSC-aCMs and iPSC-vCMs. Data are normalized to *GAPDH* levels. (C) Representative blots of cTnI and MYL2 in both types of cell lysates. β-actin served as a loading control. Experiments were performed using at least three independent biological replicates. Uncropped blots are presented in Figure S16. (D) qRT-PCR analysis of lncRNA *H19* levels in iPSC-aCMs treated with Ang II at different concentrations (1, 2.5, and 5 μM). (E) qRT-PCR analysis of lncRNA *H19* levels in cardiac tissues from patients with or without AF. Data are normalized to *GAPDH* levels. (F, G) qRT-PCR analysis of lncRNA *H19*, *ANP*, *BNP*, and *β-MHC* levels in the indicated groups. Data are normalized to *GAPDH* levels. (H, I) Representative immunofluorescence images of α-actinin (green)- and Hoechst 33342 (blue)-stained iPSC-aCMs along with quantified data showing cell surface area. Scale bar = 50 μm. Ang II was used at 2.5 μM unless otherwise indicated. *Compared with the control group, *P < 0.05, **P < 0.01, ***P < 0.001, ****P < 0.0001. ^#^Compared with the Ang II-treated group, ^#^*P* < 0.05. aCMs, iPSC-derived atrial cardiomyocytes; vCMs, iPSC-derived ventricular cardiomyocytes; Ang II, angiotensin II; NAF, patients without AF; AF, patients with AF; siNC, negative control siRNA; siH19, lncRNA *H19* siRNA.

To explore the relationship between lncRNA *H19* and cardiac hypertrophy, we first differentiated iPSCs into atrial cardiomyocytes (iPSC-aCMs) (**Figure 2A**). Compared with iPSC-vCMs, iPSC-aCMs exhibited significantly higher expression of atrial-specific genes, including *CACNA1D*, *GJA5*, *KCNA5*, *KCNJ3*, and *NPPA*, whereas ventricle-specific genes, including *HEY2*, *MYH7*, and *MYL2*, were expressed at lower levels (**Figure 2B**). Consistently, Western blot analysis demonstrated increased expression of the ventricle-specific protein MYL2 in iPSC-vCMs compared with iPSC-aCMs, confirming successful atrial differentiation (**Figure 2C** and **Figure S1A, B**). We next established an *in vitro* AF model by optimizing the concentration of Ang II to induce a hypertrophic response. Treatment of iPSC-aCMs with Ang II at different concentrations (1, 2.5, and 5 μM) for 24 h resulted in a dose-dependent upregulation of hypertrophic marker genes, including atrial natriuretic peptide (*ANP*), brain natriuretic peptide (*BNP*), and β-myosin heavy chain (*β-MHC*), as determined by qRT-PCR (**Figure S2A**). Similarly, Ang II treatment increased the cell surface area in a dose-dependent manner, as indicated by confocal images (**Figure S2B, C**). Notably, 2.5 μM of Ang II effectively induced hypertrophic responses; therefore, this concentration was selected for all subsequent functional experiments.

As shown in **Figure S3A, B**, exosomal lncRNA *H19* levels were significantly lower in the conditioned culture medium of Ang II-treated iPSC-aCMs compared with Ang II-untreated conditions. Consistently, cellular lncRNA *H19* levels were also significantly reduced in Ang II-treated iPSC-aCMs in a dose-dependent manner, indicating its potential association with cardiac hypertrophy (**Figure 2D**). In addition, lncRNA *H19* levels were markedly lower in cardiac tissues from patients with AF than in those without AF (**Figure 2E**). Importantly, these patient samples exhibited significantly elevated expression of hypertrophic markers, including *ANP*, *BNP*, and *β-MHC*, confirming the presence of hypertrophic remodeling in the clinical cohort (**Figure S4A-C**). The simultaneous elevation of hypertrophic markers and reduction of lncRNA *H19* in these tissue samples supports a link between lncRNA *H19* dysregulation and cardiac hypertrophy.

Next, we examined whether regulation of lncRNA *H19* affects Ang II-induced hypertrophic responses. In Ang II-treated iPSC-aCMs, transfection with siH19 effectively suppressed lncRNA *H19* levels by approximately 48.4%, indicating efficient knockdown (**Figure 2F**). As shown in **Figure 2G-I,** inhibition of lncRNA *H19* significantly exacerbated Ang II-induced hypertrophic responses, as evidenced by increased levels of hypertrophic markers and enlarged cell surface area. However, transfection with siNC did not affect lncRNA *H19* levels or Ang II-induced hypertrophic changes. Conversely, overexpression of lncRNA *H19* markedly increased its levels by approximately 41% and attenuated hypertrophic responses, resulting in reduced *ANP*, *BNP*, and *β-MHC* levels, as well as decreased cell surface area compared with the other groups (**Figure S5A-D**). Therefore, these findings indicate that lncRNA *H19* functions as a negative regulator of cardiac hypertrophy.

### *miR-141-3p* and *miR-200a-3p* are direct targets of lncRNA *H19*

**Figure 3:**
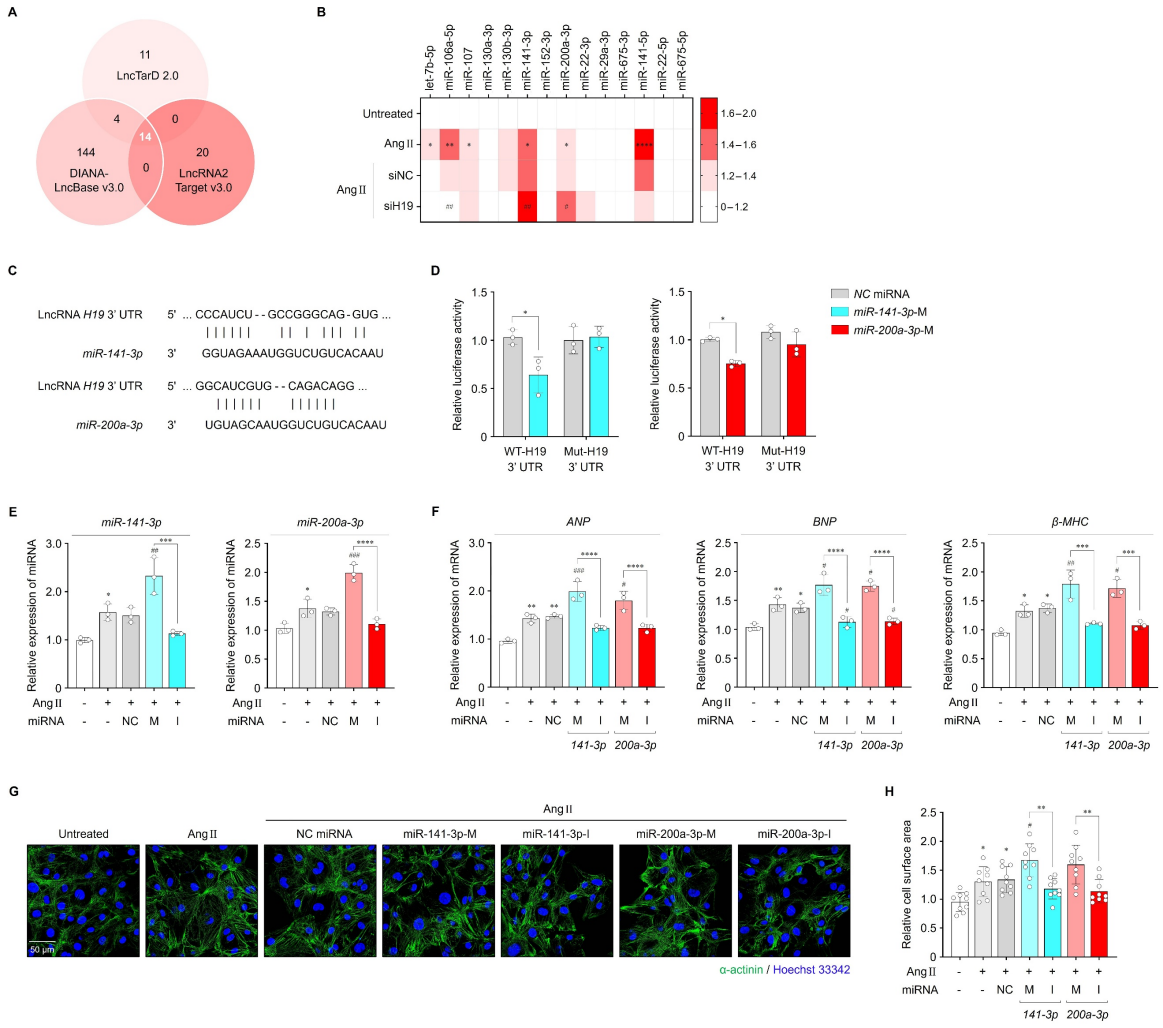
Effects of *miR-141-3p* and *miR-200a-3p* on lncRNA *H19*-mediated cardiac hypertrophy. (A) Venn diagram showing overlapping target miRNAs of lncRNA *H19* predicted using LncTarD version 2.0, DIANA-LncBase version 3.0, and LncRNA2Target version 3.0. (B) qRT-PCR analysis of the levels of 14 identified overlapping candidates in the indicated groups. Data are normalized to *U6* levels. (C) Binding site sequences of *miR-141-3p*, *miR-200a-3p*, and 3′-UTR of lncRNA *H19*. (D) Targeting relationship of lncRNA *H19* with *miR-141-3p* and *miR-200a-3p*, as determined using luciferase reporter assays. (E, F) qRT-PCR analysis of *miR-141-3p*, *miR-200a-3p*, *ANP*, *BNP*, and *β-MHC* levels in the indicated groups. Data are normalized to *U6* and *GAPDH* levels. (G, H) Representative immunofluorescence images of α-actinin (green)- and Hoechst 33342 (blue)-stained iPSC-aCMs along with quantified data showing cell surface area. Scale bar = 50 μm. Ang II was used at 2.5 μM unless otherwise indicated. *Compared with the control group, *P < 0.05, **P < 0.01, ***P < 0.001, ****P < 0.0001. ^#^Compared with the Ang II-treated group, ^#^*P* < 0.05, ^##^*P* < 0.01, ^###^*P* < 0.001. Ang II, angiotensin II; siNC, negative control siRNA; siH19, lncRNA *H19* siRNA; WT, wild-type; Mut, mutated; NC, negative control; M, mimic; I, inhibitor.

As a key player in diverse biological processes, lncRNAs regulate gene expression either positively or negatively through multiple mechanisms, such as acting signals, decoys, guides, and scaffolds 7, 33. Recently, the interaction between lncRNAs and miRNAs has emerged as an important regulatory mechanism 34. LncRNAs harboring complementary binding sites for miRNAs can regulate gene expression as competing endogenous RNAs (ceRNAs) or miRNA sponges, thereby reducing miRNA availability to target mRNAs 7. Because this regulatory network may exhibit significant impact on multiple diseases 7, 34, we focused on the lncRNA-miRNA-mRNA axis to gain insight into the molecular mechanism of lncRNA *H19*-mediated cardiac hypertrophy. Accordingly, we first analyzed the potential miRNAs interacting with lncRNA *H19* using LncTarD (version 2.0; https://lnctard.bio-database.com/), DIANA-LncBase (version 3.0; https://diana.e-ce.uth.gr/lncbasev3/), and LncRNA2Target (version 3.0; http://bio-computing.hrbmu.edu.cn/lncrna2target/index.jsp/). As shown in **Figure 3A,** fourteen miRNAs were screened as potential targets of lncRNA *H19* and are listed in **Table S7**. The levels of several miRNAs, including *miR-let-7b-5p*, *miR-106a-5p*, *miR-107*, *miR-141-3p*, *miR-200a-3p*, and *miR-141-5p*, were significantly upregulated following Ang II treatment. Notably, *miR-141-3p* and *miR-200a-3p* levels were further increased in cells transfected with siH19 compared with those transfected with siNC (**Figure 3B**). In contrast, overexpression of lncRNA *H19* significantly reduced the levels of both miRNAs (**Figure S6A**). In addition, *miR-141-3p* and *miR-200a-3p* were markedly upregulated in both cardiac tissues and serum-derived exosomes from patients with AF (**Figure S7A, B**). Therefore, *miR-141-3p* and *miR-200a-3p* were selected for subsequent mechanistic analyses.

As shown in **Figure 3C**, lncRNA *H19* contains distinct binding sites for *miR-141-3p* and *miR-200a-3p*. To determine whether *miR-141-3p* and *miR-200a-3p* directly interact with lncRNA *H19*, we constructed luciferase plasmids carrying either the WT or Mut 3′-UTR of lncRNA *H19*. Co-transfection of WT-lncRNA *H19* with *miR-141-3p* or *miR-200a-3p* mimics significantly reduced luciferase activity in HEK293 cells, whereas no change was observed with Mut-lncRNA *H19* (**Figure 3D**), indicating a direct interaction between lncRNA *H19* and *miR-141-3p* or *miR-200a-3p*. This interaction was further validated using an RNA pull-down assay. LncRNA *H19* was pulled down by Bio-WT-141 or Bio-WT-200a. In contrast, mutations in the binding sites of *miR-141-3p* and *miR-200a-3p* abolished their ability to pull down lncRNA *H19* (**Figure S8A, B**), demonstrating that *miR-141-3p* and *miR-200a-3p* bind directly to lncRNA *H19* in a sequence-specific manner. We then performed loss- and gain-of-function experiments by transfecting cells with mimics and inhibitors of *miR-141-3p* and *miR-200a-3p* to evaluate their effects on cardiac hypertrophy. After transfection with either *miR-141-3p* or *miR-200a-3p* mimics, the levels of each miRNA were significantly increased by approximately 48.3% and 45%, respectively, compared with those in Ang II-treated iPSC-aCMs (**Figure 3E**). Similarly, transfection with each miRNA inhibitor reduced the respective miRNA levels by approximately 28% and 19.4%, whereas no significant changes were observed in cells transfected with NC miRNA for either miRNA. Consequently, *miR-141-3p* or *miR-200a-3p* overexpression markedly aggravated Ang II-induced hypertrophic responses, as indicated by increased mRNA levels of hypertrophic markers and enlarged cell surface area (**Figure 3F-H**). In contrast, *miR-141-3p* or *miR-200a-3p* inhibition significantly alleviated Ang II-induced hypertrophic responses. Likewise, *miR-141-3p* and *miR-200a-3p* levels were effectively altered following transfection with their respective mimics (approximately 64.5% and 55.9%, respectively) and inhibitors (approximately 22.2% and 18.5%, respectively) under Ang II-untreated conditions, which subsequently regulated the expression of hypertrophic markers and cell surface area (**Figure S9A-D**). Therefore, these findings suggest that *miR-141-3p* and *miR-200a-3p* are direct targets of lncRNA *H19* and may be involved in lncRNA *H19*-mediated cardiac hypertrophy.

### *miR-141-3p* and *miR-200a-3p* exert their functions through PTEN

**Figure 4:**
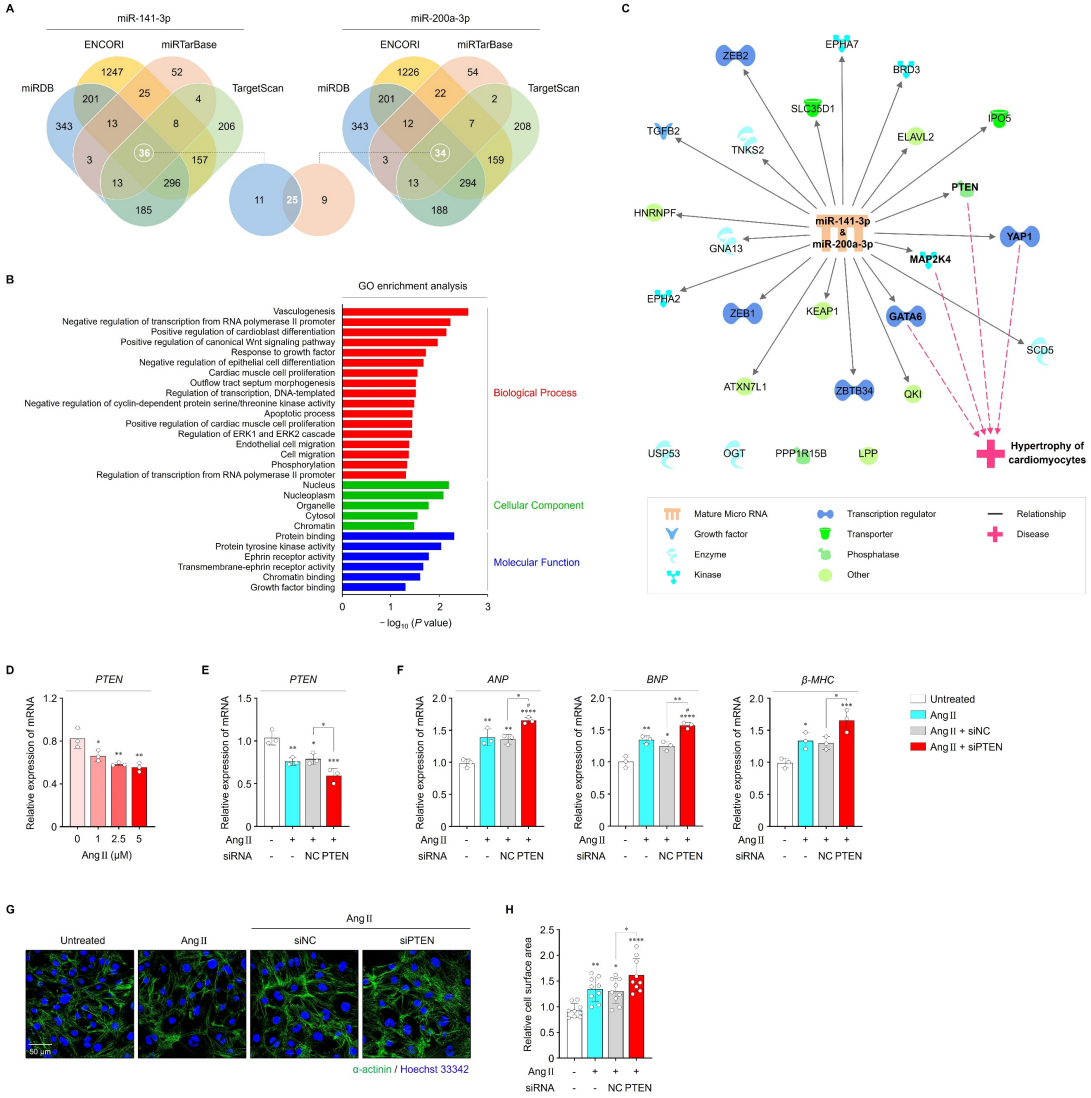
Effects of PTEN on cardiac hypertrophy. (A) Venn diagram showing overlapping target genes between *miR-141-3p* and *miR-200a-3p* predicted using diverse databases. (B, C) GO enrichment and core pathway analyses of the 25 identified overlapping candidates. (D) qRT-PCR analysis of *PTEN* levels in iPSC-aCMs treated with Ang II at different concentrations (1, 2.5, and 5 μM). (E, F) qRT-PCR analysis of *PTEN*, *ANP*, *BNP*, and *β-MHC* levels in the indicated groups. Data are normalized to *GAPDH* levels. (G, H) Representative immunofluorescence images of α-actinin (green)- and Hoechst 33342 (blue)-stained iPSC-aCMs along with quantified data showing cell surface area. Scale bar = 50 μm. Ang II was used at 2.5 μM unless otherwise indicated. *Compared with the control group, *P < 0.05, **P < 0.01, ***P < 0.001, ****P < 0.0001. ^#^Compared with the Ang II-treated group, ^#^*P* < 0.05. GO, gene ontology; Ang II, angiotensin II; siNC, negative control siRNA; siPTEN, *PTEN* siRNA.

To explore the downstream signaling mechanisms of *miR-141-3p* and *miR-200a-3p* in regulating cardiac hypertrophy, we predicted the target genes of both miRNAs using multiple computational databases, including miRDB, ENCORI, miRTarBase, and TargetScan. Venn diagram analysis identified 25 overlapping candidate target genes for both miRNAs (**Figure 4A**). GO enrichment analyses of biological processes, cellular components, and molecular functions were then conducted to investigate the potential roles of these genes. Notably, GO biological process analysis revealed significant enrichment in terms of positive regulation of cardioblast differentiation, positive regulation of the canonical WNT signaling pathway, response to growth factor, cardiac muscle cell proliferation, apoptotic processes, regulation of ERK1 and ERK2 cascade, and cell migration, all of which are associated with cardiac hypertrophic remodeling (**Figure 4B**). Among the 25 genes, core pathway analysis indicated that *PTEN*, *YAP1*, *MAP2K4*, and *GATA6* may be involved in cardiac hypertrophy (**Figure 4C**). In addition, PPI network analysis highlighted PTEN as a key hub gene (**Figure S10A**). PTEN has been widely reported as a critical regulator of cardiac hypertrophy, eventually leading to AF 35, 36. Previous studies have demonstrated that cardiomyocyte-specific deletion of PTEN leads to hypertrophic cardiomyopathy, and that PTEN inhibition elicits cardiac hypertrophy through interruption of PINK1-AMPK signaling and autophagy 37, 38. Based on these findings, PTEN was selected as a potential downstream candidate mediating the hypertrophic effects of *miR-141-3p* and *miR-200a-3p*.

In cardiac tissues from patients with AF, *PTEN* levels were significantly reduced compared with those from patients without AF (**Figure S11A**). In parallel, Ang II treatment resulted in a dose-dependent decrease in *PTEN* mRNA levels in iPSC-aCMs, which were further suppressed by approx.imately 21.7% following transfection with *PTEN* siRNA (**Figure 4D, E**). Inhibition of *PTEN* markedly exacerbated Ang II-induced hypertrophic responses, as indicated by increased levels of hypertrophic markers (*ANP*, *BNP*, and *β-MHC*) and enlarged cell surface area compared with the other groups (**Figure 4F-H**). Therefore, these findings imply that PTEN may contribute to cardiac hypertrophy through its interaction with *miR-141-3p* and *miR-200a-3p*.

### LncRNA *H19* mediates cardiac hypertrophy through miR-141-3p and miR-200a-3p/PTEN pathway

**Figure 5:**
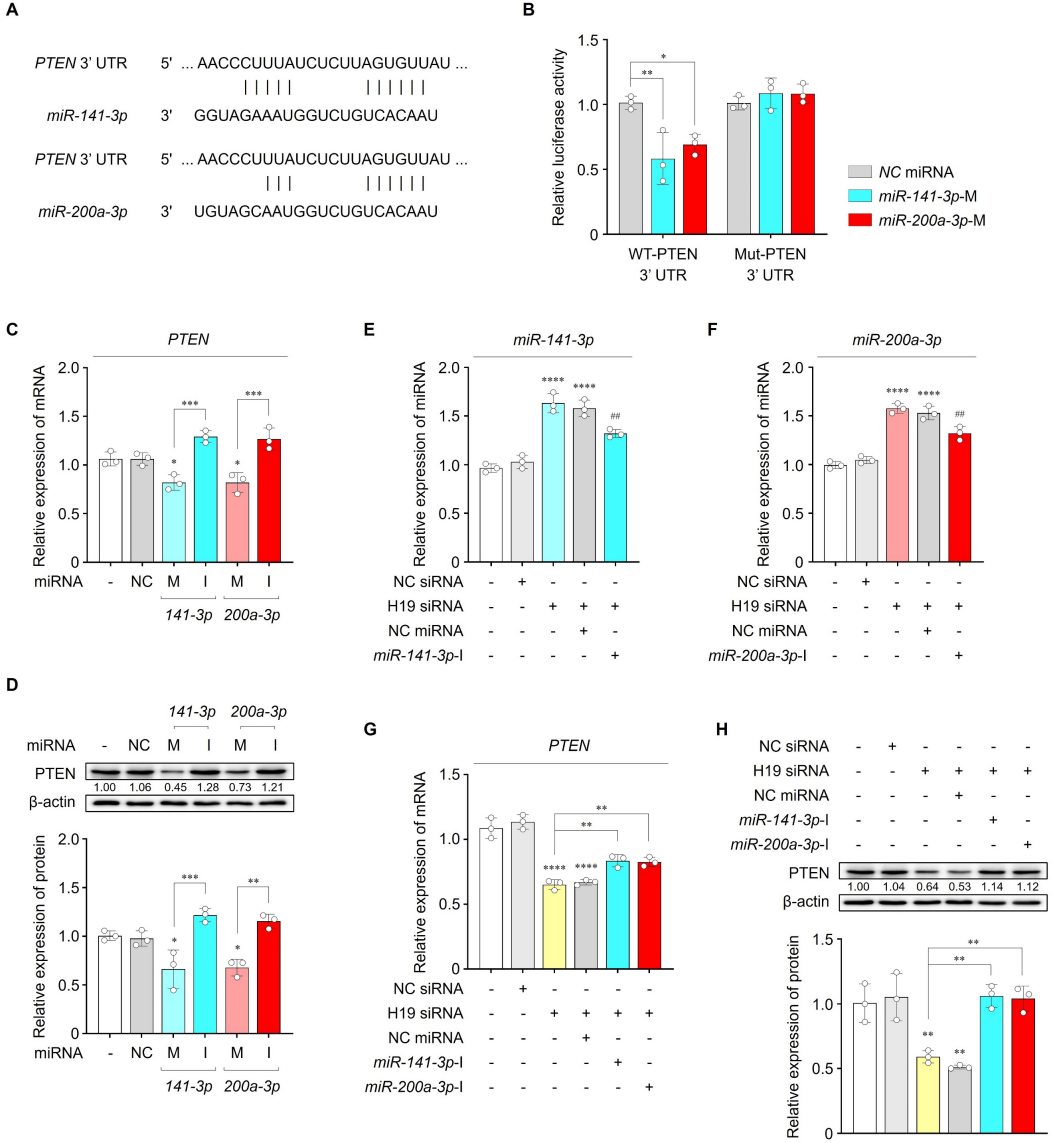
LncRNA *H19*-mediated cardiac hypertrophy through the miR-141-3p and miR-200a-3p/PTEN pathway. (A) Binding site sequences of *miR-141-3p*, *miR-200a-3p*, and 3′-UTR of *PTEN*. (B) Targeting relationship of *miR-141-3p* and *miR-200a-3p* with *PTEN*, as determined using luciferase reporter assays. (C) qRT-PCR analysis of *PTEN* levels in the indicated groups. Data are normalized to *GAPDH* levels. (D) Representative blots and quantified data showing PTEN levels in the indicated groups. β-actin served as a loading control. Experiments were performed using at least three independent biological replicates. Uncropped blots are shown in Figure S16. (E**-**G) qRT-PCR analysis of *miR-141-3p*, *miR-200a-3p*, and *PTEN* levels in the indicated groups. Data are normalized to *U6* and *GAPDH* levels. (H) Representative blots and quantified data showing protein levels in the indicated groups. β-actin served as a loading control. Experiments were performed using at least three independent biological replicates. Uncropped blots are shown in Figure S16. *Compared with the control group, *P < 0.05, **P < 0.01, ***P < 0.001, ****P < 0.0001. ^#^Compared with the siH19-transfected group, ^##^*P* < 0.01. WT, wild-type; Mut, mutated; NC, negative control; M, mimic; I, inhibitor.

After confirming *miR-141-3p* and *miR-200a-3p* binding sites at the *PTEN* 3′-UTR, we constructed luciferase plasmids containing either the WT or Mut 3′-UTR of *PTEN* (**Figure 5A**). Co-transfection of HEK293 cells with WT-*PTEN* and *miR-141-3p* or *miR-200a-3p* mimics resulted in a significant reduction in luciferase activity, whereas no significant change was observed with Mut-*PTEN* (**Figure 5B**). Additionally, mRNA and protein levels of PTEN were significantly decreased by *miR-141-3p* or *miR-200a-3p* mimics and were increased by their respective inhibitors in both untreated and Ang II-treated iPSC-aCMs (**Figure 5C, D** and **Figure S12A-C**). Therefore, these findings demonstrate that PTEN is a direct downstream target of *miR-141-3p* and *miR-200a-3p*.

We further examined whether *miR-141-3p* and *miR-200a-3p*, together with their target PTEN, mediate the action of lncRNA *H19* on cardiac hypertrophy. As shown in **Figure 5E-H,** inhibition of lncRNA *H19* resulted in increased *miR-141-3p* and *miR-200a-3p* levels, along with decreased PTEN expression at both the mRNA and protein levels. However, these effects were rescued by co-transfection with *miR-141-3p* or *miR-200a-3p* inhibitors, respectively. Collectively, these findings indicate that lncRNA *H19* regulates cardiac hypertrophy by acting as a ceRNA for *miR-141-3p* and *miR-200a-3p*, thereby modulating PTEN expression.

### LncRNA *H19* attenuates cardiac hypertrophy *in vivo*

**Figure 6:**
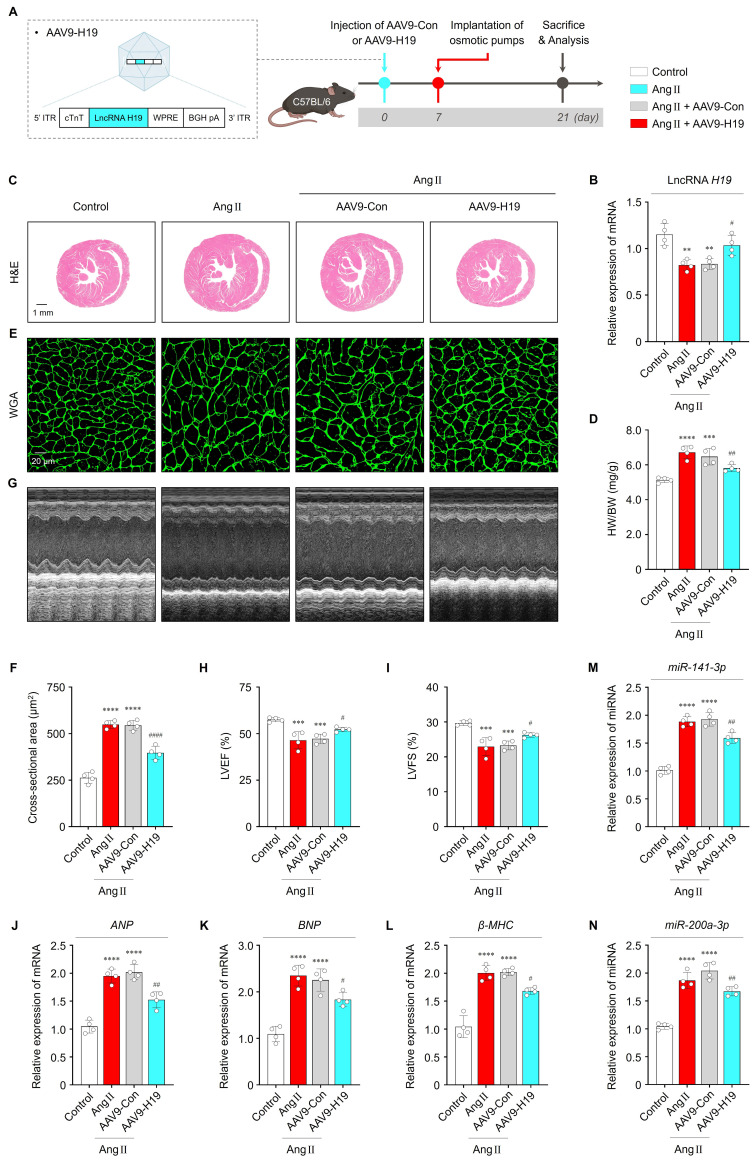
Therapeutic effects of lncRNA *H19 in vivo*. (A) Schematic illustration of the AAV9-H19 construct and *in vivo* experimental procedure. (B) qRT-PCR analysis of lncRNA *H19* levels in the indicated groups (n = 4 per group). Data are normalized to *GAPDH* levels. (C, D) Representative images of H&E-stained cardiac sections and quantified data showing HW/BW (n = 4 per group). Scale bar = 1 mm. (E, F) Representative immunofluorescence images of wheat germ agglutinin (WGA)-stained cardiac sections and quantified data showing cell surface area (n = 4 per group). Scale bar = 20 μm. (G**-**I) Representative M-mode images and quantified data showing LVEF and LVFS in the indicated groups (n = 4 per group). (J**-**N) qRT-PCR analysis of *ANP*, *BNP*, *β-MHC*, *miR-141-3p*, and *miR-200a-3p* levels in the indicated groups (n = 4 per group). Data are normalized to *GAPDH* and *U6* levels. *Compared with the control group, **P < 0.01, ***P < 0.001, ****P < 0.0001. ^#^Compared with the Ang II-treated group, ^#^*P* < 0.05, ^##^*P* < 0.01, ^####^*P* < 0.0001. ITR, inverted terminal repeat; WPRE, woodchuck hepatitis virus posttranscriptional regulatory element; BGH pA, bovine growth hormone polyadenylation signal; AAV9-Con, empty control adeno-associated virus serotype 9 vector; AAV9-H19, adeno-associated virus serotype 9 expressing lncRNA *H19*; Ang II, angiotensin II; HW/BW, heart weight-to-body weight; LVEF, left ventricular ejection fraction; LVFS, left ventricular fractional shortening.

To evaluate the effects of lncRNA *H19* on cardiac hypertrophy *in vivo*, mice were intravenously injected with AAV9-Con or AAV9-H19, followed by implantation 7 days later of Ang II-containing micro-osmotic pumps (or PBS-containing pumps as a control) for 2 weeks (**Figure 6A**). As shown in **Figure 6B**, lncRNA *H19* levels were significantly increased in the cardiac tissues of AAV9-H19-injected mice compared with those of AAV9-Con-injected mice. Importantly, viral-mediated overexpression of lncRNA *H19* did not affect key biochemical parameters, including ALT, AST, BUN, CRE, and LDH, indicating *in vivo* safety (**Figure S13A-E**). Consequently, cardiac-specific overexpression of lncRNA *H19* markedly ameliorated Ang II-induced cardiac hypertrophy, as demonstrated by a reduced heart weight-to-body weight (HW/BW) ratio and decreased cell surface area compared with the other groups (**Figure 6C-F**). In addition, echocardiography showed that cardiac functional parameters were significantly improved in AAV9-H19-injected mice (**Figure 6G-I**). Consistent with these structural and functional improvements, Ang II-induced increases in *ANP*, *BNP*, and *β-MHC* levels were significantly reversed by AAV9-H19 injection (**Figure 6J-L**). As shown in **Figure 6M** and** N,** lncRNA *H19* overexpression resulted in decreased levels of *miR-141-3p* and *miR-200a-3p*. Moreover, immunofluorescence and Western blot analyses revealed increased PTEN expression in the cardiac tissues of AAV9-H19-injected mice (**Figure S14A, B** and **Figure S15A, B**). Given that PTEN is a major negative regulator of AKT signaling 39, we further examined whether lncRNA *H19* overexpression suppresses AKT activation *in vivo*. In AAV9-H19-injected mice, Ang II-induced AKT phosphorylation was significantly attenuated compared with the other groups (**Figure S15A, C**). Collectively, these findings suggest that lncRNA *H19* exerts cardioprotective effects *in vivo* through the miR-141-3p and miR-200a-3p/PTEN/AKT signaling pathway, supporting its potential therapeutic relevance in cardiac hypertrophy.

## Discussion

In the present study, we demonstrate that lower lncRNA *H19* levels in serum exosomes are closely linked with AF, suggesting its potential as a diagnostic biomarker. In addition, our findings indicate that lncRNA *H19* is functionally involved in cardiac hypertrophy, highlighting its therapeutic relevance in AF. Mechanistically, lncRNA *H19* deficiency promotes hypertrophic responses through upregulation of *miR-141-3p* and *miR-200a-3p*, resulting in suppression of the PTEN/AKT pathway.

Numerous studies have identified exosomal lncRNAs as molecular signatures of their parent cells, exhibiting enhanced stability and disease-specific expression patterns compared with non-exosomal lncRNAs 7, 20. Importantly, the lipid bilayer membrane of exosomes protects their molecular content from enzymatic degradation in circulation 20, 21, thereby supporting the use of exosome-based diagnostic approaches rather than cell-free circulating lncRNAs. In this study, serum was selected as the source of circulating exosomes because it is a commonly used matrix in clinical laboratories due to its ease of preparation and handling, absence of anticoagulants-which can interfere with routine blood tests and immunoassays-and the availability of long-established reference standards 40. However, it is recognized that the presence of platelet-derived vesicles in serum may influence exosome profiles 41, suggesting the need for further studies to validate their diagnostic applicability. Nevertheless, specific lncRNAs depleted or enriched in serum-derived exosomes have gained increasing attention as noninvasive diagnostic biomarkers for cardiovascular diseases, including atherosclerosis (exosomal lncRNA *PELATON*) and acute myocardial infarction (exosomal *HOTAIR*) 42, 43. However, a limited number of studies have investigated the role of serum exosomal lncRNAs in predicting and diagnosing AF 44. Herein, we identified distinct changes in serum exosomal lncRNA profiles between patients with and without AF using RNA sequencing analysis. Notably, lncRNA *H19* was consistently downregulated in both cardiac tissues and serum-derived exosomes in patients with AF compared with those without AF. Moreover, serum exosomal lncRNA *H19* levels were negatively correlated with LAD and demonstrated significant diagnostic performance, supporting the clinical relevance of lncRNA *H19* in AF. Although lncRNA *H19* has also been reported in other cardiovascular conditions, accumulating evidence suggests that the direction and regulatory mechanisms of lncRNA *H19* differ across cardiovascular diseases 45-48. In this study, we focused on the characteristic expression pattern of lncRNA *H19* in serum exosomes of patients with AF and its functional roles in AF pathogenesis, rather than emphasizing exclusive disease specificity. Considering that the pathophysiology of AF is complex and multifactorial, the decrease in exosomal lncRNA *H19* levels may, at least in part, reflect broader cardiac pathologies such as cardiac hypertrophy or remodeling. Nevertheless, further validation through prospective and multicenter studies is warranted to enhance its diagnostic utility. Therefore, our findings highlight serum exosomal lncRNA *H19* as a promising diagnostic biomarker for AF.

AF is recognized as a progressive arrhythmia resulting from cumulative structural and electrical remodeling 15, 49. Notably, cardiac hypertrophy is a well-known risk factor for AF 30. Clinically, AF is the most common sustained arrhythmia in hypertrophic cardiomyopathy, affecting one in five patients, among whom AF is associated with a markedly increased risk of stroke 50. Accordingly, numerous studies have focused on the prevention of cardiac hypertrophy as a potential upstream therapeutic strategy for AF 51, 52. However, effective treatments to alleviate cardiac hypertrophy remain limited 53. Recent studies have reported that certain lncRNAs are implicated in cardiac hypertrophy, highlighting their potential as therapeutic targets for AF 54. In this study, we demonstrated that both loss- and gain-of-function of lncRNA *H19* exert significant pro- and anti-hypertrophic effects, respectively, in Ang II-treated iPSC-aCMs and mice. Consistently, a previous study showed that lncRNA *H19* functions as a negative regulator of cardiac hypertrophy in a transverse aortic constriction mouse model 55. Moreover, cardiomyocyte-directed gene therapy using murine and human lncRNA *H19* has been shown to markedly attenuate heart failure, even in the presence of cardiac hypertrophy 48. Although atrial-specific pathological changes (e.g., atrial fibrosis, AF inducibility) were not assessed in AAV9-H19-injected mice, we confirmed that lncRNA *H19* overexpression significantly reduced hypertrophic markers and improved cardiac function. These findings suggest a protective role of lncRNA *H19* against upstream remodeling processes that contribute to the development and progression of AF. Therefore, targeting lncRNA *H19*-mediated cardiac hypertrophy may represent a promising therapeutic approach for AF.

The lncRNA-miRNA-mRNA axis modulates diverse biological functions in different diseases 34, 56. However, the molecular mechanisms of cardiac hypertrophy based on these regulatory networks remain poorly understood. Our findings identified *miR-141-3p* and *miR-200a-3p* as direct targets of lncRNA *H19*, exhibiting a negative correlation with its expression. Specifically, upregulation of either *miR-141-3p* or *miR-200a-3p* exacerbated hypertrophic responses, whereas their downregulation significantly attenuated these outcomes. Mechanistically, *miR-141-3p* and *miR-200a-3p* exerted their effects by directly binding to the 3′-UTR of *PTEN*, leading to suppression of PTEN expression and subsequent promotion of cardiac hypertrophy in both untreated and Ang II-treated iPSC-aCMs. Consistent with previous studies highlighting the critical role of PTEN in cardiac hypertrophy 37, 38, 57, PTEN inhibition in our study further aggravated Ang II-induced hypertrophic responses. Importantly, suppression of lncRNA *H19* reduced PTEN expression through its interaction with *miR-141-3p* and *miR-200a-3p*, thereby promoting cardiac hypertrophy. Given that the PTEN/AKT pathway is a major signaling axis involved in the pathogenesis of cardiac hypertrophy 39, our *in vivo* analyses demonstrated that lncRNA *H19* overexpression markedly suppressed Ang II-induced AKT phosphorylation. These findings implicate PTEN/AKT signaling as a key downstream mediator of the anti-hypertrophic effects of lncRNA *H19*. Overall, our study demonstrates that lncRNA *H19* functions as a key regulator of cardiac hypertrophy through the miR-141-3p and miR-200a-3p/PTEN/AKT axis, providing mechanistic insight into AF-associated hypertrophic remodeling and highlighting lncRNA *H19* as a potential therapeutic target for AF.

Although circulating exosomal lncRNAs have been considered important factors in various diseases, their uncertain cellular origin remains a major barrier to clinical translation 58-60. In cardiovascular diseases, accumulating evidence indicates that long-distance intercellular communication is mediated by exosomes secreted from different cardiac cell types into the systemic circulation, thereby facilitating functional crosstalk between the heart and other organs (e.g., bone marrow) 59, 61-63. Accordingly, the most likely mechanism is that cardiac cell type-specific exosomes containing functional biomolecules, such as lncRNAs, are released into the bloodstream and subsequently detected as being either downregulated or upregulated in patients with AF compared with those without AF. In exosomes derived from Ang II-treated iPSC-aCMs-conditioned culture medium, we observed significantly lower levels of lncRNA *H19* compared with Ang II-untreated conditions. Given that cardiomyocytes are the most prevalent cardiac cell type 64, these findings suggest that cardiomyocytes may represent a potential cellular source of exosomal lncRNA *H19*. Nevertheless, the origin of circulating exosomal lncRNA *H19* from other cardiac (e.g., fibroblasts, endothelial cells) or non-cardiac cell types cannot be excluded; therefore, future studies clarifying the cellular origin would strengthen its translational significance.

Despite great promise of exosome-based diagnostics and therapeutics across a wide range of diseases, several translational challenges remain for the clinical application of serum exosomal lncRNA *H19*. From a diagnostic perspective, the lack of standardized protocols for exosome isolation and characterization represents a major barrier 65. Despite multiple isolation techniques, there is currently no universally accepted reference standard 65, 66. Importantly, isolating exosomes is often associated with a series of challenges that significantly impact purity, yield, and overall sample quality, thereby compromising the reliability of exosome-based biomarker measurements due to variability in pre-analytical and analytical procedures 65-67. Therefore, systematic clinical validation studies with standardized protocols are essential to establish assay sensitivity and specificity, as well as robust and reproducible cut-off values, ultimately improving diagnostic accuracy and supporting broader clinical adoption. From a therapeutic perspective, although AAV9-mediated gene delivery has demonstrated efficacy in preclinical models, key concerns remain regarding delivery efficiency, safety, immunogenicity, and potential off-target effects 68, 69. Moreover, given the chronic nature of AF 2, ensuring durable and long-term stable therapeutic effects will require optimization of nanotechnology-based delivery platforms and dosing strategies for lncRNA *H19*-based interventions. Collectively, continued integrative efforts will be necessary to fully realize the clinical potential of exosomes in both diagnostic and therapeutic applications.

The present study has several limitations. First, the sample sizes of cardiac tissues and RNA-sequenced serum exosomes were not sufficiently large to confirm AF-specific lncRNAs. Therefore, further studies with larger cohorts will be necessary to validate the clinical relevance of exosomal lncRNA *H19*. Second, considering the diversity and complexity of lncRNA targets, the miR-141-3p and miR-200a-3p/PTEN axis may represent one of multiple pathways that regulate cardiac hypertrophy. Therefore, further investigations-including comprehensive exosomal mRNA profiling, systematic analyses of exosome-mediated ceRNA regulatory networks, and tissue-level spatial analyses-will be important for providing in-depth mechanistic and translational insights. Third, although Ang II-treated mice have been widely used as a reproducible *in vivo* model for mechanistic studies of AF pathogenesis, this model may not fully reflect AF pathophysiology 35, 70, 71; thus, further studies using suitable preclinical models will be required to elucidate the functional role of lncRNA *H19* in AF and to validate its potential as a genetic or pharmacological therapeutic target.

In conclusion, our findings not only identify lncRNA *H19* as a potential theragnostic target for AF but also elucidate a key molecular mechanism by which lncRNA *H19* regulates cardiac hypertrophy, thereby providing novel insights into the prevention and treatment of AF.

## Supplementary Material

Supplementary figures and tables.

## Data Availability

The datasets used in the study are available from the corresponding author on reasonable request.

## Abbreviations

AF: atrial fibrillation
LncRNAs: long noncoding RNAs
iPSC: induced pluripotent stem cell
aCMs: atrial cardiomyocytes
vCMs: ventricular cardiomyocytes
Ang II: angiotensin II
NTA: nanoparticle tracking analysis
UTR: untranslated region
qRT-PCR: quantitative reverse transcription polymerase chain reaction
GO: gene ontology
PPI: protein-protein interaction
AAV9: adeno-associated virus serotype 9
LVEF: left ventricular ejection fraction
LVFS: left ventricular fractional shortening
ALT: alanine aminotransferase
AST: aspartate aminotransferase
BUN: blood urea nitrogen
CRE: creatinine
LDH: lactate dehydrogenase
LAD: left atrial diameter
ROC: receiver operating characteristic
AUC: area under the curve
CI: confidence interval
ANP: atrial natriuretic peptide
BNP: brain natriuretic peptide
β-MHC: β-myosin heavy chain
ceRNAs: competing endogenous RNAs
