# Supplementary material for: Exosomal Long Noncoding RNA H19 as a Biomarker and Therapeutic Target in Atrial Fibrillation
Source: Int J Biol Sci. 2026 Mar 17;22(7):3470–87. doi: 10.7150/ijbs.123108 (PMC13085887; doi:10.7150/ijbs.123108)
Supplement: Supplementary file 1 — Supplementary figures and tables. [file ijbsv22p3470s1.pdf]

Supplementary Material

Exosomal Long Noncoding RNA H19 as a Biomarker and Therapeutic Target in Atrial Fibrillation

Ji-Young Kang[1], Dasom Mun[1], Malgeum Park[1], Gyeongseo Yoo[2], Nuri Yun[3,], Boyoung Joung[1,2,*]*

[1]Division of Cardiology, Yonsei University College of Medicine, 50-1 Yonsei-ro, Seodaemun-gu, Seoul 03722, Republic of Korea; [2]Graduate School of Medical Science, Brain Korea 21 Project, Yonsei University College of Medicine, 50-1 Yonsei-ro, Seodaemun-gu, Seoul 03722, Republic of Korea; and [3]GNTPharma Science and Technology Center for Health, 85 Songdogwahak-ro, Yeonsu-gu, Incheon 21983, Republic of Korea

***Corresponding authors**:
Boyoung Joung
Division of Cardiology, Yonsei University College of Medicine, Seoul 03722, Republic of Korea
Tel: +82-2-2228-8447, Fax: +82-2-2227-7732
Email: cby6908@yuhs.ac

Nuri Yun
GNTPharma Science and Technology Center for Health, Incheon 21983, Republic of Korea
Tel: +82-31-8005-9910, Fax: +82-31-8005-9917
Email: yunnuri@hanmail.net

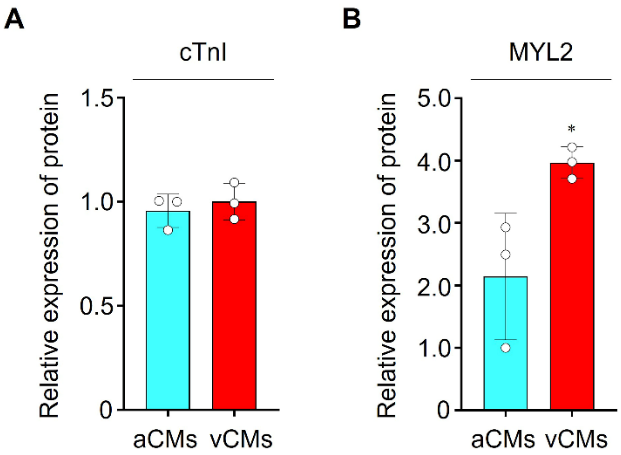

Figure S1. Western blot analysis of iPSC-aCMs and iPSC-vCMs. (A, B) Quantified data corresponding to the representative blots shown in Figure 2C. β-actin served as a loading control. Experiments were performed using at least three independent biological replicates. *$P < 0.05$ compared with aCMs. aCMs, iPSC-derived atrial cardiomyocytes; vCMs, iPSC-derived ventricular cardiomyocytes.

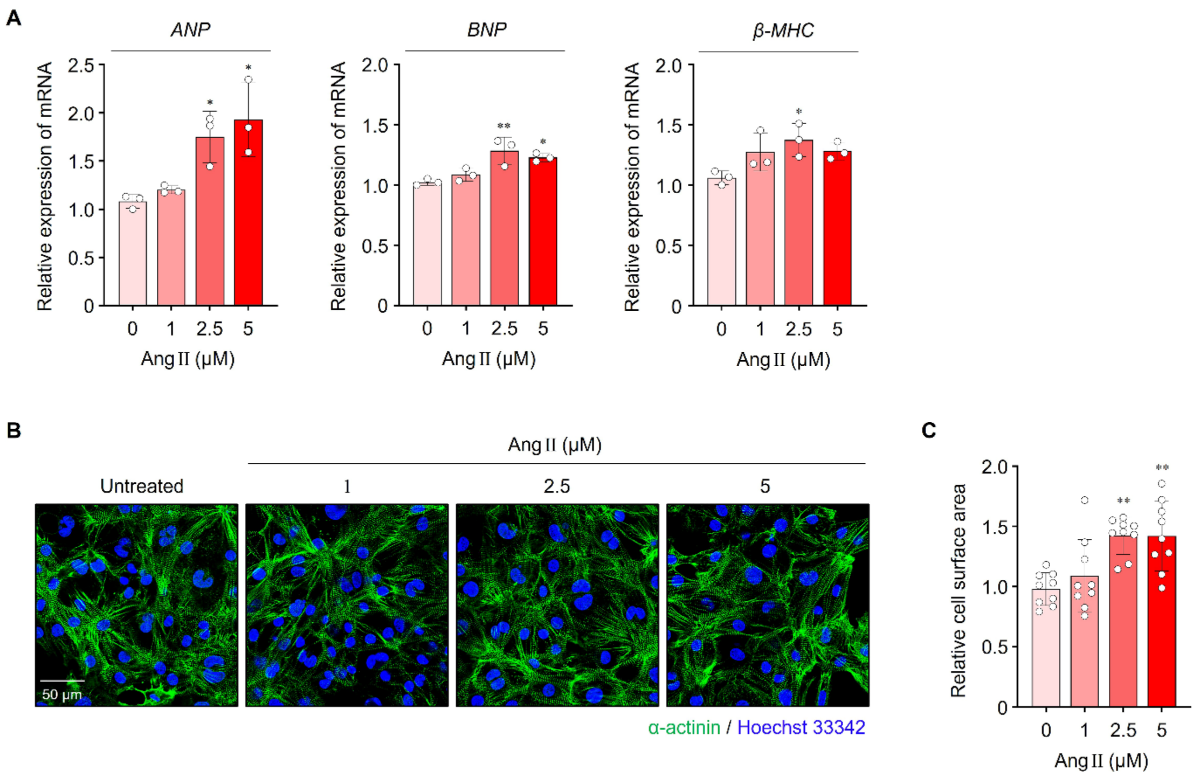

Figure S2. Effects of Ang II on cardiac hypertrophy. (A) qRT-PCR analysis of *ANP*, *BNP*, and *β-MHC* levels in iPSC-aCMs treated with Ang II at different concentrations (1, 2.5, and 5μM). Data are normalized to *GAPDH* levels. (B, C) Representative immunofluorescence images of α-actinin (green)- and Hoechst 33342 (blue)-stained iPSC-aCMs along with quantified data showing cell surface area. Scale bar = 50 μm. *Compared with the control group, *$P < 0.05$, **$P < 0.01$. Ang II, angiotensin II.

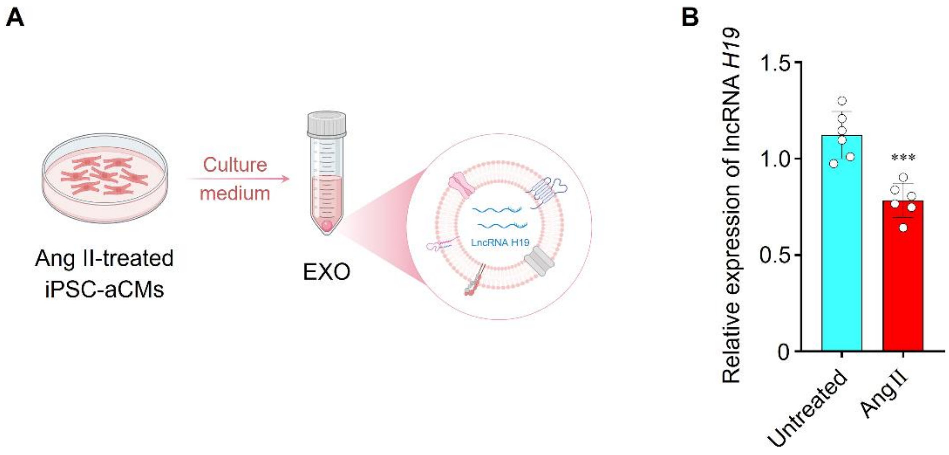

Figure S3. Expression levels of lncRNA *H19* in exosomes derived from culture medium. (A, B) qRT-PCR analysis of exosomal lncRNA *H19* levels in conditioned culture medium from untreated and Ang II-treated iPSC-aCMs. Data are normalized to *GAPDH* levels. *Compared with the control group, ***$P < 0.001$. iPSC-aCMs, iPSC-derived atrial cardiomyocytes; Ang II, angiotensin II.

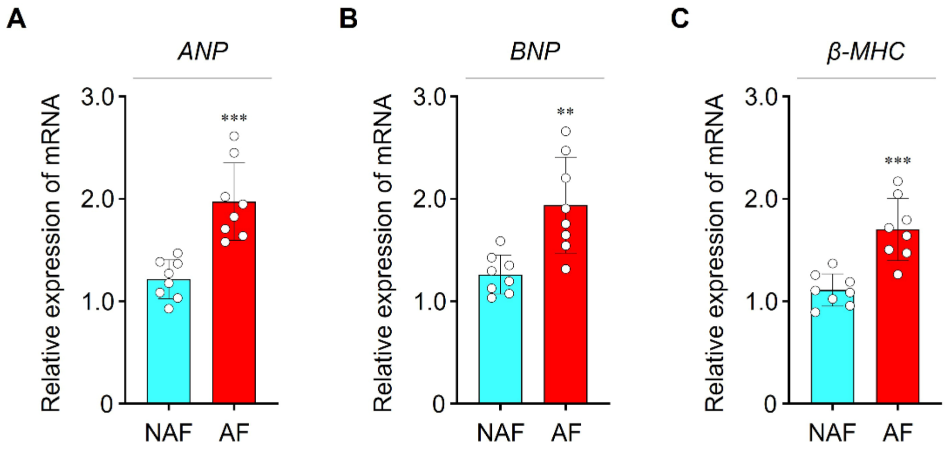

Figure S4. Expression levels of hypertrophic marker genes in human cardiac tissues. (A–C) qRT-PCR analysis of *ANP*, *BNP*, and *β-MHC* levels in cardiac tissues from patients with or without AF. Data are normalized to *GAPDH* levels. *Compared with the control group, $**P < 0.01$, $***P < 0.001$. NAF, patients without AF; AF, patients with AF.

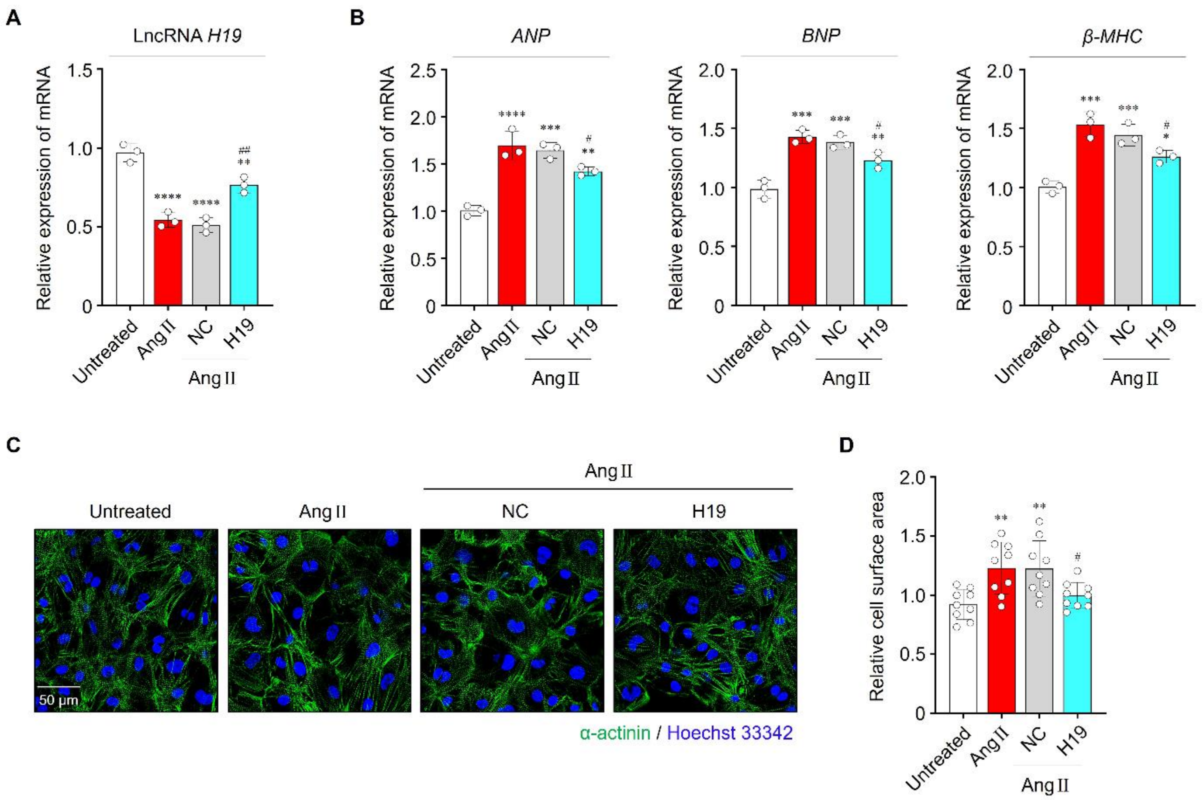

Figure S5. Effects of lncRNA *H19* overexpression on cardiac hypertrophy. (A, B) qRT-PCR analysis of lncRNA *H19*, *ANP*, *BNP*, and *β-MHC* levels in the indicated groups. Data are normalized to *GAPDH* levels. (C, D) Representative immunofluorescence images of α-actinin (green)- and Hoechst 33342 (blue)-stained iPSC-aCMs along with quantified data showing cell surface area. Scale bar = 50 μm. *Compared with the control group, *$P < 0.05$, **$P < 0.01$, ***$P < 0.001$, ****$P < 0.0001$. #Compared with the Ang II-treated group, #$P < 0.05$, ##$P < 0.01$. Ang II was used at 2.5 μM unless otherwise indicated. Ang II, angiotensin II; NC, negative control vector; H19, lncRNA *H19* overexpression vector.

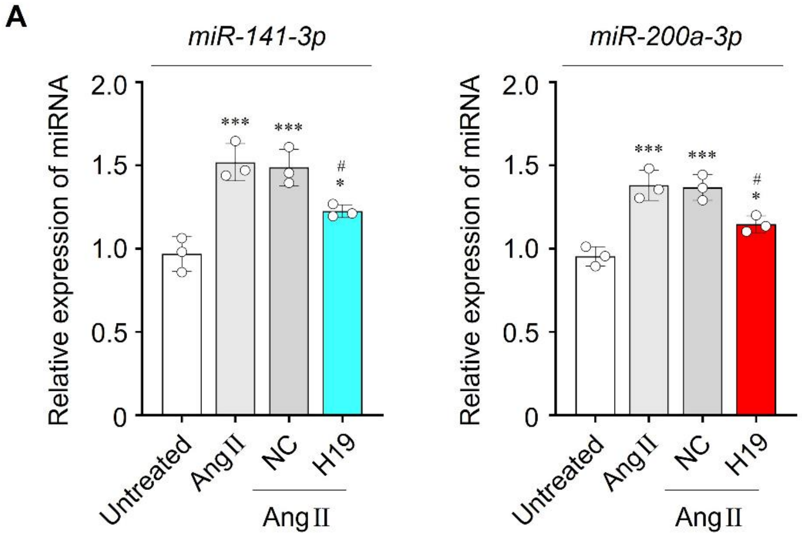

Figure S6. Effects of lncRNA *H19* overexpression on miRNA levels. (A) qRT-PCR analysis of *miR-141-3p* and *miR-200a-3p* levels in the indicated groups. Data are normalized to *U6* levels. *Compared with the control group, *P < 0.05, ***P < 0.001. #Compared with the Ang II-treated group, #P < 0.05. Ang II was used at 2.5 μM unless otherwise indicated. Ang II, angiotensin II; NC, negative control vector; H19, lncRNA *H19* overexpression vector.

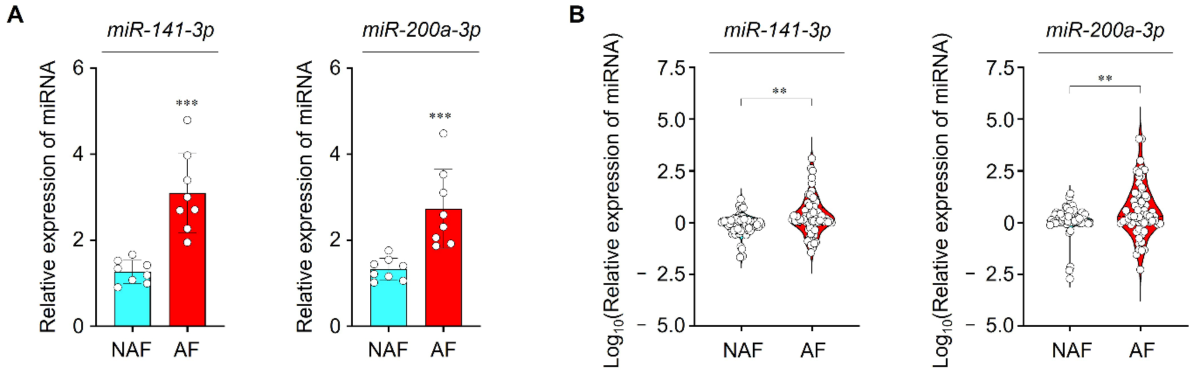

Figure S7. Expression levels of *miR-141-3p* and *miR-200a-3p* in different sample types. (A, B) qRT-PCR analysis of *miR-141-3p* and *miR-200a-3p* levels in cardiac tissues and serum exosomes from patients with AF. Data are normalized to *U6* levels. *Compared with the control group, $^{**}P < 0.01$, $^{***}P < 0.001$.

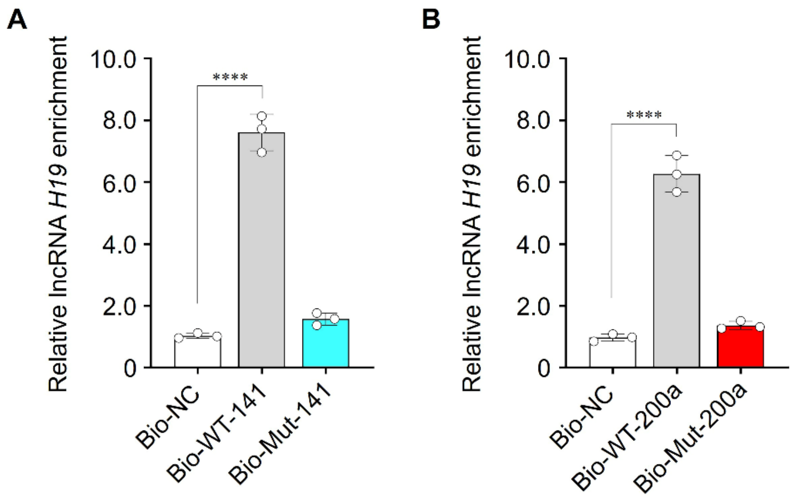

Figure S8. Identification of the interaction between lncRNA *H19* and *miR-141-3p* or *miR-200a-3p*. (A, B) Biotin-labeled miRNA pull-down assay followed by qRT-PCR analysis of lncRNA *H19* enrichment in the indicated groups. Data are normalized to *GAPDH* levels. *Compared with the control group, ****P < 0.0001. Bio-NC, biotin-labeled negative control; Bio-WT-141, biotin-labeled wild-type-*miR-141-3p*; Bio-Mut-141, biotin-labeled mutated-*miR-141-3p*; Bio-WT-200a, biotin-labeled wild-type-*miR-200a-3p*; Bio-Mut-200a, biotin-labeled mutated-*miR-200a-3p*.

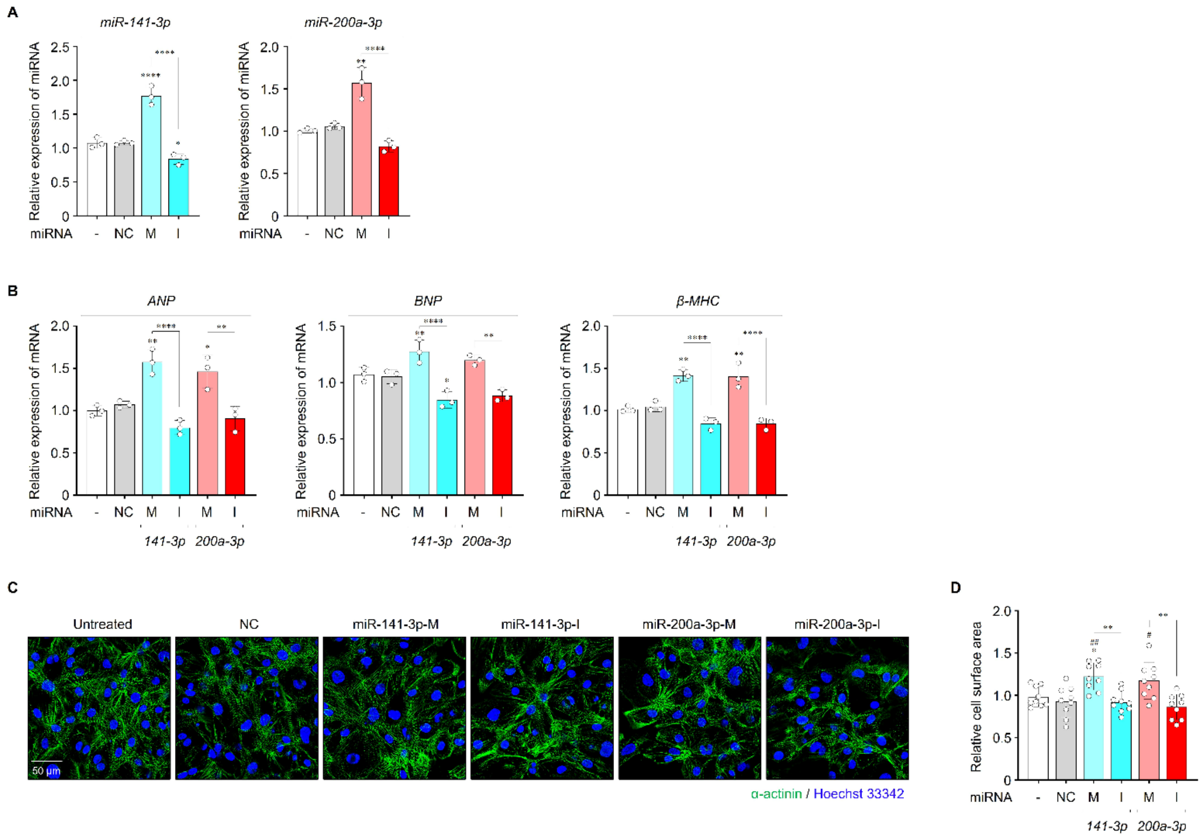

Figure S9. Effects of *miR-141-3p* and *miR-200a-3p* on cardiac hypertrophy. (A, B) qRT-PCR analysis of *miR-141-3p*, *miR-200a-3p*, *ANP*, *BNP*, and *β-MHC* levels in the indicated groups. Data are normalized to *U6* and *GAPDH* levels. (C, D) Representative immunofluorescence images of α-actinin (green)- and Hoechst 33342 (blue)-stained iPSC-aCMs along with quantified data showing cell surface area. Scale bar = 50 μm. *Compared with the control group, *$P < 0.05$, **$P < 0.01$, ****$P < 0.0001$. #Compared with the NC miRNA-transfected group, #$P < 0.05$, ##$P < 0.01$. NC, negative control; M, mimic; I, inhibitor.

A

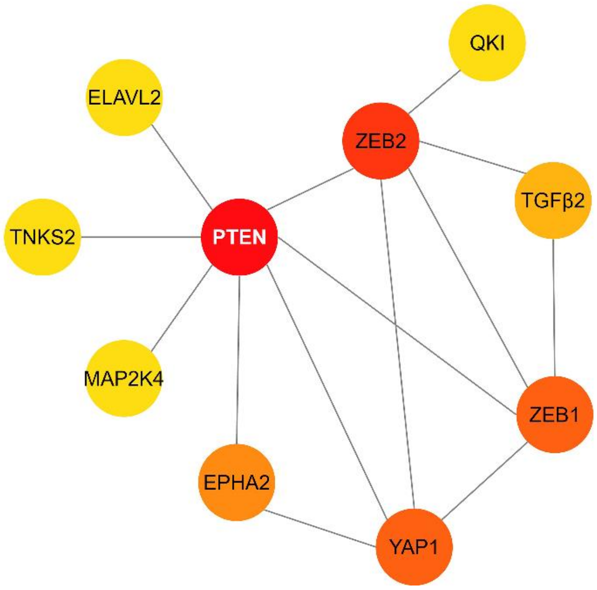

Figure S10. PPI network analysis. (A) Hub genes identified using the CytoHubba plug-in of Cytoscape based on the maximal clique centrality algorithm.

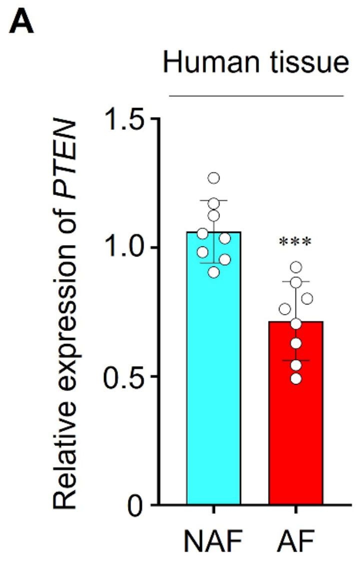

Figure S11. Expression levels of *PTEN* in human cardiac tissues. (A) qRT-PCR analysis of *PTEN* levels in cardiac tissues from patients with or without AF. Data are normalized to *GAPDH* levels. *Compared with the control group, ***$P < 0.001$. NAF, patients without AF; AF, patients with AF.

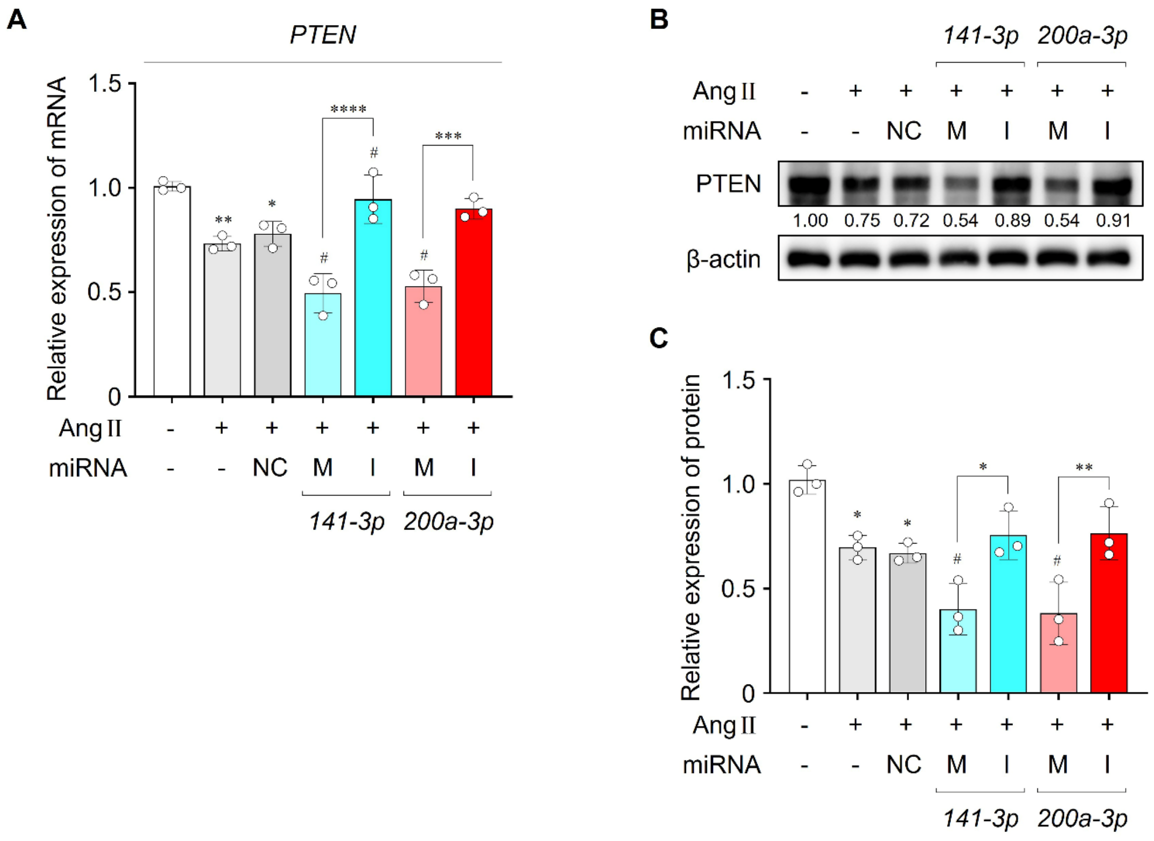

Figure S12. Effects of *miR-141-3p* and *miR-200a-3p* on PTEN expression in Ang II-treated iPSC-aCMs. (A) qRT-PCR analysis of *PTEN* levels in the indicated groups. Data are normalized to *GAPDH* levels. (B, C) Representative blots and quantified data showing PTEN levels in the indicated groups. β-actin served as a loading control. Experiments were performed using at least three independent biological replicates. Uncropped blots are shown in Figure S16. *Compared with the control group, *$P < 0.05$, **$P < 0.01$, ***$P < 0.001$, ****$P < 0.0001$. #Compared with the Ang II-treated group, #$P < 0.05$. Ang II was used at 2.5 μM unless otherwise indicated. Ang II, angiotensin II; NC, negative control; M, mimic; I, inhibitor.

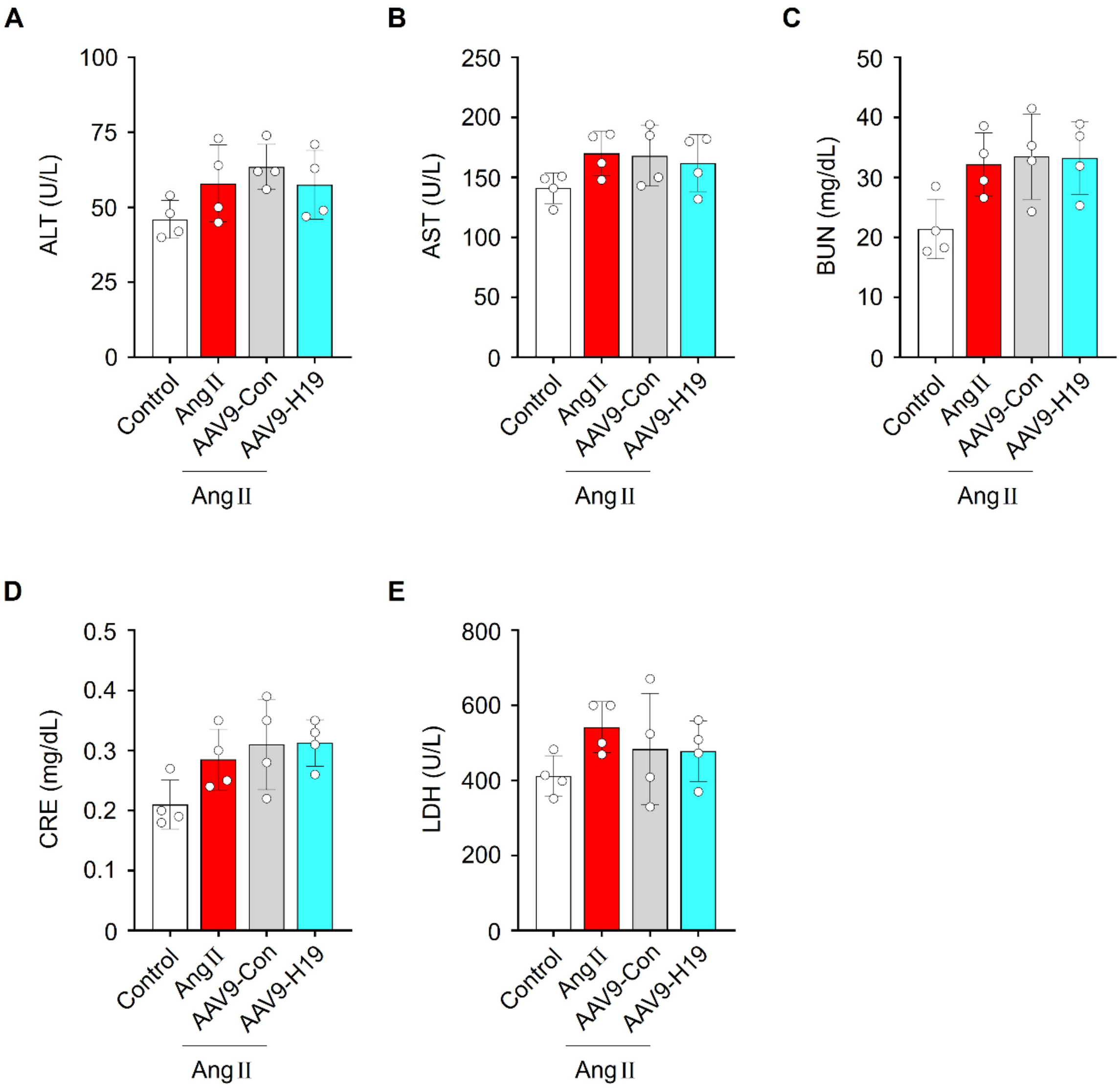

Figure S13. *In vivo* biosafety of AAV9-mediated overexpression of lncRNA *H19*. (A–E) The levels of alanine aminotransferase (ALT), aspartate aminotransferase (AST), blood urea nitrogen (BUN), creatinine (CRE), and lactate dehydrogenase (LDH) in the indicated groups (n = 4 per group). AAV9-Con, empty control adeno-associated virus serotype 9 vector; AAV9-H19, adeno-associated virus serotype 9 expressing lncRNA *H19*; Ang II, angiotensin II.

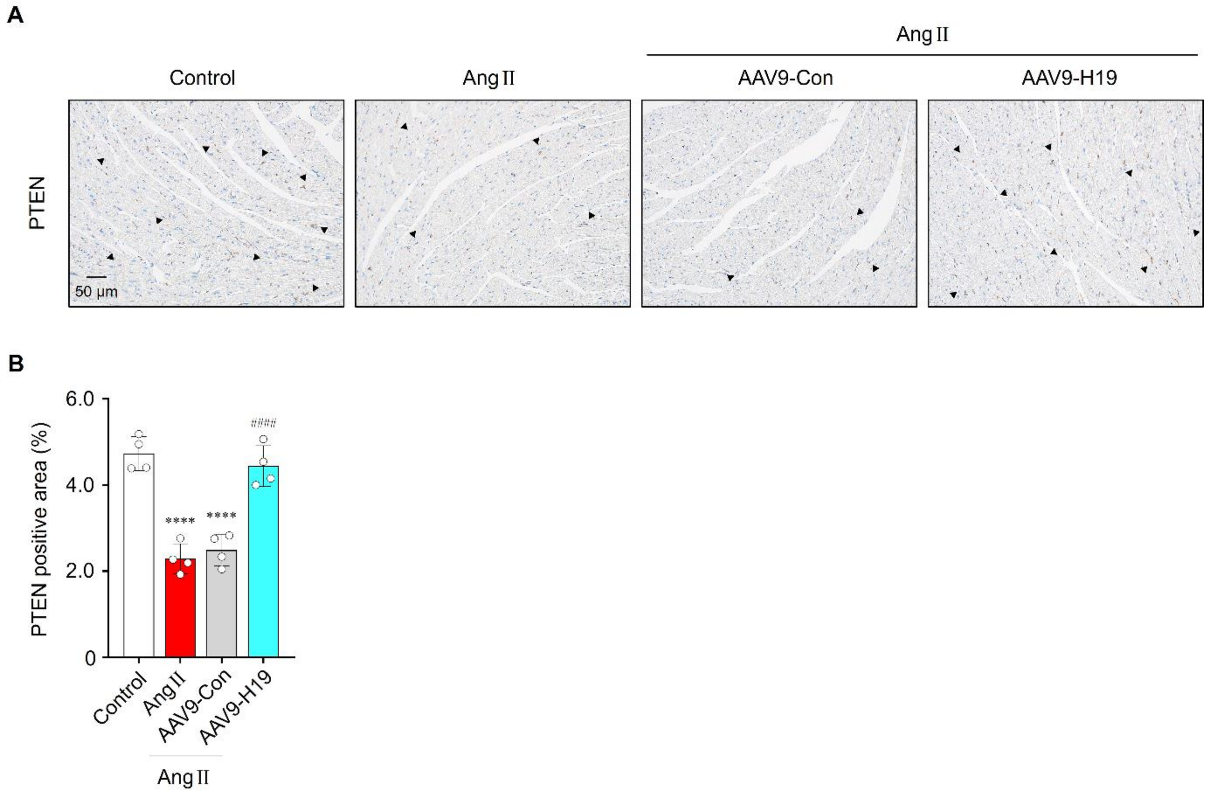

Figure S14. Effects of lncRNA *H19* on PTEN levels in Ang II-treated mice. (A, B) Representative images of PTEN-stained cardiac sections and quantified data showing PTEN positive area (%) (n = 4 per group). Scale bar = 50 μm. *Compared with the control group, ****$P < 0.0001$. #Compared with the Ang II-treated group, ####$P < 0.0001$. AAV9-Con, empty control adeno-associated virus serotype 9 vector; AAV9-H19, adeno-associated virus serotype 9 expressing lncRNA *H19*; Ang II, angiotensin II.

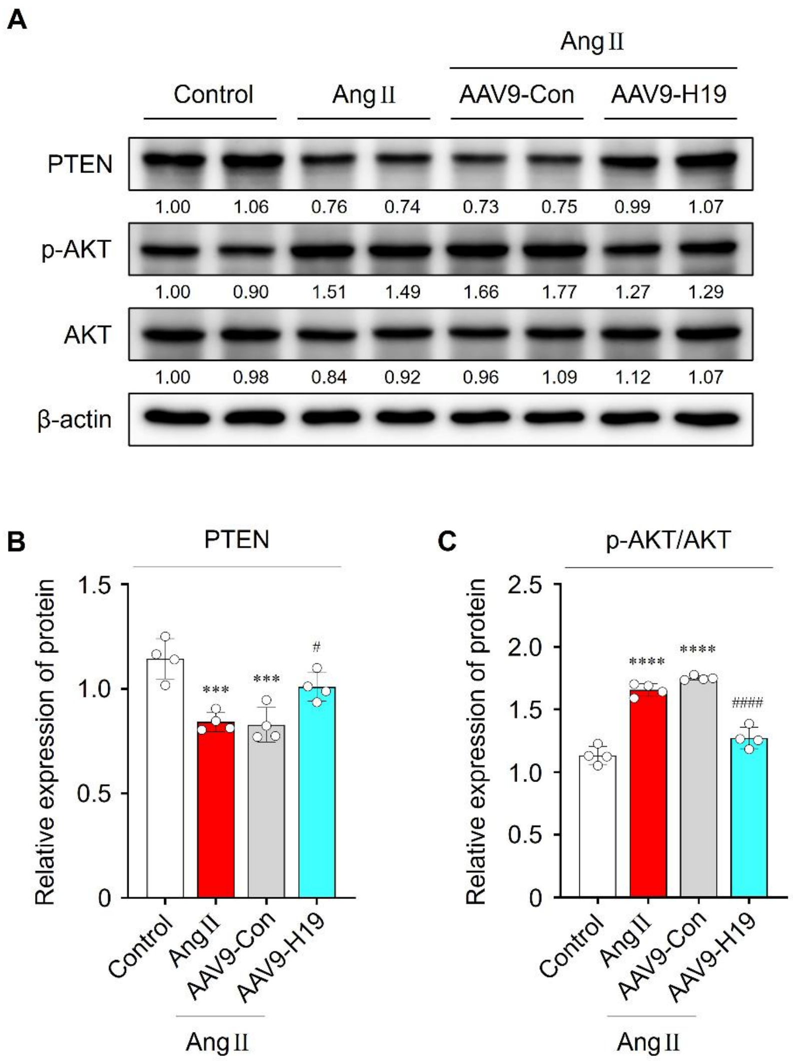

Figure S15. Effects of lncRNA *H19* on PTEN/AKT signaling in Ang II-treated mice. (A–C) Representative blots and quantified data showing protein levels in the indicated groups. β-actin served as a loading control. Experiments were performed using at least three independent biological replicates. Uncropped blots are shown in Figure S16. *Compared with the control group, ***$P < 0.001$, ****$P < 0.0001$. #Compared with the Ang II-treated group, #$P < 0.05$, ####$P < 0.0001$. AAV9-Con, empty control adeno-associated virus serotype 9 vector; AAV9-H19, adeno-associated virus serotype 9 expressing lncRNA *H19*; Ang II, angiotensin II.

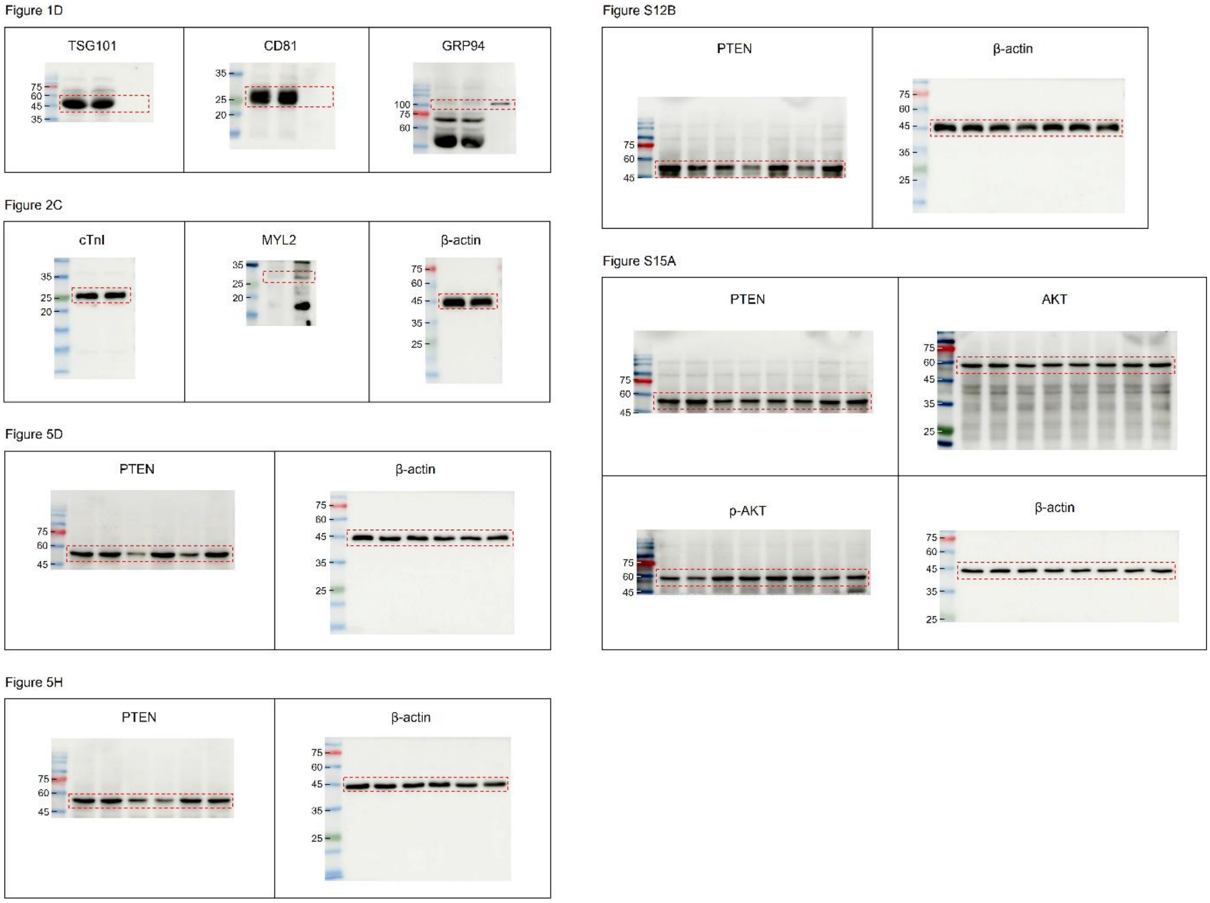

Figure S16. Uncropped blots corresponding to Figure 1D, Figure 2C, Figure 5D, Figure 5H, Figure S12B, and Figure S15A. Red boxes indicate the cropped regions shown in the respective figures.

Table S1. Clinical profiles of patients from whom serum samples were obtained.

Variables	RNA sequencing			qRT-PCR		
	NAF (n = 3)	AF (n = 3)	P value	NAF (n = 68)	AF (n = 68)	P value
Age, years	56 (54.5–58)	56 (55.5–57)	> 0.999	57.5 (53–61.3)	58.5 (51.8–63.3)	> 0.999
Male, n (%)	2 (67)	2 (67)	1.000	44 (65)	53 (78)	0.129
BMI (kg, m^2)	22 (21.8–24.8)	26.4 (25.3–26.4)	0.434	25 (23–26.3)	25.6 (24.2–28)	0.003
Coronary artery disease, n (%)	0 (0)	0 (0)	N/A	1 (1)	2 (3)	> 0.999
Hypertension, n (%)	1 (33)	2 (67)	0.414	21 (31)	29 (43)	0.213
Diabetes mellitus, n (%)	1 (33)	1 (33)	1.000	6 (9)	13 (19)	0.136
Stroke, n (%)	0 (0)	0 (0)	N/A	0 (0)	0 (0)	N/A
Systolic BP (mm Hg)	137 (133–148.5)	131 (124–155)	0.988	128 (118.8–139)	133 (121.8–145)	0.065
Diastolic BP (mm Hg)	90 (90–93)	74 (70.5–86)	0.318	78 (69.5–81.3)	89 (80–94.3)	< 0.001
LAD (mm)	29 (27.5–32.5)	43 (42.5–48)	0.028	35 (32–39)	43 (40–47)	< 0.001
LVEF (%)	65 (64.5–70.5)	62 (60–65)	0.309	67 (63.8–72)	62 (56–67)	< 0.001
E/E'	8 (7.5–9)	8 (7–8)	0.420	9 (7–10.3)	9 (7–10)	0.988

Values are expressed as n (%) or median (interquartile range).

BMI, body mass index; BP, blood pressure; LAD, left atrial diameter; LVEF, left ventricular ejection fraction.

Table S2. Clinical profiles of patients from whom tissue samples were obtained.

Variables	NAF (n = 8)	AF (n = 8)	P value
Age, years	65.5 (62.8–67.8)	65.5 (64–68.5)	0.882
Male, n (%)	5 (63)	5 (63)	> 0.999
BMI (kg, m^2)	23.6 (21.4–25)	22.1 (20.1–26.3)	0.962
Coronary artery disease, n (%)	1 (13)	1 (13)	> 0.999
Hypertension, n (%)	5 (63)	3 (38)	0.619
Diabetes mellitus, n (%)	1 (13)	4 (50)	0.282
Stroke, n (%)	0 (0)	0 (0)	N/A
Systolic BP (mm Hg)	128 (106.8–139.3)	95.5 (90–101)	0.145
Diastolic BP (mm Hg)	69.5 (62–74)	68 (58.3–75.5)	0.967
LAD (mm)	36.5 (32.8–41.3)	50 (46–52.3)	< 0.001
LVEF (%)	65 (60–70)	50 (34–61.3)	0.139
E/E'	12.9 (10.5–15.4)	14 (12.6–17.9)	0.878

Values are expressed as n (%) or median (interquartile range).

BMI, body mass index; BP, blood pressure; LAD, left atrial diameter; LVEF, left ventricular ejection fraction.

Table S3. List of biotinylated miRNAs used for pull-down assay.

Gene	Sequence (5’-3’)
NC	UUCUCCGAACGUGUCACGUtt
WT-miR-141-3p	UAACACUGUCUGGUAAAGAUGG
Mut-miR-141-3p	UAACACUGUCUGGUAAUCUACC
WT-miR-200a-3p	UAACACUGUCUGGUAACGAUGU
Mut-miR-200a-3p	UAACACAGACAGGUAUCCAAGU

NC, negative control; WT, wild-type; Mut, mutated.

Table S4. List of primers used for qRT-PCR.

Gene	Forward sequence (5'-3')	Reverse sequence (5'-3')
H19 (human)	AGACAGTACAGCATCCAGGG	GAGACCTGGCCTCGTCTC
ANP (human)	CAGGATGGACAGGATTGGA	TGTCCTCCCTGGCTGTTATC
BNP (human)	TCAGCCTCGGACTTGGAAAC	CTTCCAGACACCTGTGGGAC
β-MHC (human)	CCAAGTTCACTCACATCCATCA	AGTGGCAATAAAAGGGGTAGC
PTEN (human)	TGAGTTCCCTCAGCCGTTACCT	GAGGTTTCCTCTGGTCCTGGTA
CACNA1D (human)	CTTCGACAACGTCCTCTCTGCT	GCCGATGTTCTCTCCATTCGAG
GJA5 (human)	CCACAGAGAAGAATGTCTTCA	TGCTGCTGGCCTTACTAAGA
KCNA5 (human)	ACAGGATTGGAGCCCAGAG	GGAGCCTCTTGCAGTCTGTC
KCNJ3 (human)	GCACGCGGTGATCTCCATGA	ACCCTCAGGTGTCTGCCGA
NPPA (human)	ACAGACGTAGGCCAAGAGAG	GTCTGACCTAGGAGCTGGAA
HEY2 (human)	CTGAGTTGAGAAGACTTGTGCCAA	TGGCAAGAGCGTGTGCGTCAAA
MYH7 (human)	AAAGAGGCGCTAGAGAAGTCCG	CAGCATCTGCCAGGTTGTCTTG
MYL2 (human)	GGCGAGTGAACGTGAAAAAT	CAGCATTTCCCGAACGTAAT
GAPDH (human)	GGAGCGAGATCCCTCCAAAAT	GGCTGTTGTCATACTTCTCATGG
H19 (mouse)	TCATCATCTCCCTCCTGTCT	GGTAAATGGGGAAACAGAGT
ANP (mouse)	CTTCCTCGTCTTGGCCTTT	CCAGGTGGTCTAGCAGGTTC
BNP (mouse)	TGGGAGGTCACTCCTATCCT	GGCCATTTCCTCCGACTTT
β-MHC (mouse)	CGGACCTTGGAAGACCAGAT	GACAGCTCCCCATTCTCTGT
GAPDH (mouse)	ACAGCAACAGGGTGGTGGAC	TTTGAGGGTGCAGCGAACTT
miR-141-3p	TAACACTGTCTGGTAAAGATGG	
miR-200a-3p	TAACACTGTCTGGTAACGATGT	
let-7b-5p	TGAGGTAGTAGGTTGTGTGGTT	
miR-106a-5p	AAAAGTGCTTACAGTGCAGGTAG	
miR-107	AGCAGCATTGTACAGGGCTATCA	
miR-130a-3p	CAGTGCAATGTTAAAAGGGCAT	
miR-130b-3p	CAGTGCAATGATGAAAGGGCAT	
miR-152-3p	TCAGTGCATGACAGAACTTGG	
miR-22-3p	AAGCTGCCAGTTGAAGAACTGT	
miR-29a-3p	TAGCACCATCTGAAATCGGTTA	
miR-675-3p	CTGTATGCCCTCACCGCTCA	
miR-141-5p	CATCTTCCAGTACAGTGTTGGA	
miR-22-5p	AGTTCTTCAGTGGCAAGCTTTA	
miR-675-5p	TGGTGCGGAGAGGGCCCACAGTG	

U6	CTCGCTTCGGCAGCACA

Table S5. List of 27 differentially expressed serum exosomal lncRNAs (|fold change| ≥ 2; $P < 0.05$) in patients with AF.

High expression			Low expression		
LncRNA	Fold Change	*P* value	LncRNA	Fold Change	*P* value
LOC105373037	2.93	0.028	LOC112268071	−16.20	< 0.001
LINC01102	2.77	0.029	LOC105369890	−15.61	< 0.001
LOC107985299	2.56	0.040	LOC105373484	−12.77	0.001
LOC107987000	2.19	0.047	LOC105375928	−12.35	0.002
			LINC01783	−10.89	0.002
			LOC105371225	−9.95	0.007
			LOC105369304	−9.59	< 0.001
			BLACAT1	−7.72	0.001
			LOC105375779	−7.48	0.003
			LOC107986437	−7.12	0.001
			LOC105371767	−6.98	0.001
			H19	−6.78	0.012
			LOC107985246	−6.33	0.008
			PCED1B-AS1	−4.68	0.024
			FAM27E3	−4.45	0.012
			LOC105373709	−4.28	0.020
			SUCLG2-AS1	−3.50	0.038
			LOC105371922	−3.30	0.047
			LOC105370363	−3.20	0.047
			LINC01283	−2.94	0.012
			LOC105379185	−2.92	0.010
			LUCAT1	−2.78	0.044
			LOC105378415	−2.32	0.040

Table S6. Univariate and multivariate logistic regression analyses of serum exosomal lncRNA *H19* in patients with AF.

	Univariate			Multivariate		
Variables	OR	95% CI	*P* value	OR[a]	95% CI	*P* value
Age, years	1.00	0.96–1.05	1.000	1.05	0.97–1.14	0.212
Male, n (%)	1.78	0.85–3.84	0.129	1.15	0.33–4.17	0.827
BMI (kg, m^2)	1.22	1.07–1.40	0.003	1.04	0.80–1.34	0.775
Coronary artery disease, n (%)	1.69	0.22–18.85	0.610			
Hypertension, n (%)	1.65	0.82–3.34	0.158			
Diabetes mellitus, n (%)	2.34	0.88–6.78	0.088			
Systolic BP (mm Hg)	1.02	1.00–1.04	0.066			
Diastolic BP (mm Hg)	1.08	1.05–1.12	< 0.001	1.07	1.02–1.13	0.002
LAD (mm)	1.34	1.22–1.50	< 0.001	1.30	1.15–1.51	< 0.001
LVEF (%)	0.87	0.82–0.92	< 0.001	0.88	0.80–0.95	0.002
E/E'	1.00	0.92–1.09	0.989			
LncRNA H19	0.02	< 0.01–0.08	< 0.001	0.04	< 0.01–0.35	< 0.001

CI, confidence interval; OR, odds ratio; OR[a], adjusted odds ratio; other abbreviations as in Tables S1 and S2.

[a] Multivariate logistic regression model adjusted for age, sex, and baseline differences (BMI, coronary artery disease, diastolic BP, LAD, and LVEF).

Table S7. List of overlapping target miRNAs of lncRNA *H19*.

LncRNA	Target miRNAs
H19	let-7b-5p, miR-106a-5p, miR-107, miR-130a-3p, miR-130b-3p, miR-141-3p, miR-141-5p, miR-152-3p, miR-200a-3p, miR-22-3p, miR-22-5p, miR-29a-3p, miR-675-3p, miR-675-5p
